# TIA1‐Mediated Stress Granules Promote the Neuroinflammation and Demyelination in Experimental Autoimmune Encephalomyelitis through Upregulating IL‐31RA Signaling

**DOI:** 10.1002/advs.202409086

**Published:** 2025-01-13

**Authors:** Xin Hua, Lingting Jin, Zheyu Fang, Yiyun Weng, Yuan Zhang, Jingjing Zhang, Dewei Xie, Yang Tang, Siyu Guo, Yingying Huang, Yilin Dai, Jia Li, Zhihui Huang, Xu Zhang

**Affiliations:** ^1^ Department of Neurology The First Affiliated Hospital of Wenzhou Medical University Wenzhou Zhejiang 325000 China; ^2^ Department of Neurology Xuanwu Hospital Capital Medical University National Center for Neurological Disorders Beijing 100053 China; ^3^ School of Basic Medical Sciences Wenzhou Medical University Wenzhou Zhejiang 325000 China; ^4^ Cancer Institute, Second Affiliated Hospital, Zhejiang University School of Medicine Zhejiang University Hangzhou 310058 China; ^5^ School of Pharmacy Hangzhou Normal University Hangzhou Zhejiang 311121 China

**Keywords:** experimental autoimmune encephalomyelitis, multiple sclerosis, neuroinflammation, stress granules, T‐cell intracellular antigen 1

## Abstract

The dysfunction of stress granules (SGs) plays a crucial role in the pathogenesis of various neurological disorders, with T cell intracellular antigen 1 (TIA1) being a key component of SGs. However, the role and mechanism of TIA1‐mediated SGs in experimental autoimmune encephalomyelitis (EAE) remain unclear. In this study, upregulation of TIA1, its translocation from the nucleus to the cytoplasm, and co‐localization with G3BP1 (a marker of SGs) are observed in the spinal cord neurons of EAE mice. Deletion of TIA1 in the CNS alleviates neuroinflammation, suppresses demyelination and axonal damage, and reduces neuronal loss in EAE mice. Furthermore, alleviation of autophagy dysfunction and reduction of chronic persistent SGs are observed in *Tia1* ^Nestin^‐CKO EAE mice. Mechanistically, IL‐31RA levels are decreased in *Tia1* ^Nestin^‐CKO EAE mice, which inhibit the downstream PI3K/AKT signaling pathway associated with IL‐31RA, thereby enhancing autophagy and suppressing the NF‐κB signaling pathway, further alleviating EAE symptoms. Knockdown of TIA1 in primary neurons and N2a cells treated with sodium arsenite also reduces the formation of SGs. These findings reveal an unrecognized role of TIA1‐mediated SGs in promoting neuroinflammation and demyelination, offering novel therapeutic targets for MS.

## Graphical abstract

In EAE mice, TIA1 is translocated from the nucleus to the cytoplasm, facilitating SGs formation in neurons. TIA1 knockout in the central nervous system (CNS) of EAE mice yields a notable diminution in neuronal SGs, mitigates CNS inflammatory infiltration, and curtails axonal demyelination. Mechanistically, CNS‐specific TIA1 knockout engenders a reduction in IL‐31RA expression within spinal cord neurons.

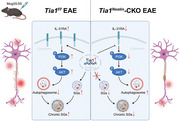

## 1 Introduction

Multiple sclerosis (MS) is an autoimmune disease of the central nervous system (CNS), marked by diffuse and focal areas of inflammation, demyelination, gliosis, and neuronal injury.^[^ ^1^, ^2^ ^]^ The cause of MS is multifactorial and is probably the cumulative result of multiple genetic and environmental risk factors. The cellular and molecular mechanisms underlying MS pathogenesis and progression are unclear. As a result, no definitive and effective treatment is available in clinical practice. Several mechanisms have been proposed, including mitochondrial dysfunction, oxidative stress, neuroinflammation, and dysfunction of RNA binding proteins (RBPs).^[^ ^3^ ^]^ Recent accumulating evidence suggests that the pathogenesis of neurological diseases is associated with the dysfunction of RBPs, RNA granules, and stress granules (SGs).^[^ ^4^ ^]^ Gaining insight into these inherent processes is key to discovering markers that can facilitate detection and intervention for the progressive stages of MS.^[^ ^5^ ^]^

Dysfunction of SGs is recognized as one of the pathological factors in neurodegenerative diseases.^[^ ^4^ ^]^ SGs, a type of membraneless organelle composed of mRNA and RBPs, primarily originate from stalled pre‐initiation translation complexes. Key components of SGs include T‐cell intracellular antigen 1 (TIA‐1), the eukaryotic translation initiation factor 2 subunit alpha (eIF2α) and rasGTPase‐activating protein‐binding protein 1 (G3BP1).^[^ ^6^, ^7^ ^]^ Previously, the formation of SGs was considered a cellular adaptive defense mechanism, protecting cells from apoptosis under adverse conditions by regulating mRNA translation and sequestering signaling molecules.^[^ ^8^ ^]^ However, recent studies have shown that neuronal death in neurological diseases is closely related to the accumulation of SGs in the cytoplasm.^[^ ^9^, ^10^ ^]^ Although SGs were considered transient structures, prolonged stress can lead to their persistence. This persistence appears to be a focal point for disease‐related protein aggregation. When SGs become persistent, the inherent fragility of cellular RNA metabolism may lead to the pathological aggregation of RBPs, thereby disrupting neuronal homeostasis and ultimately interfering with the protein quality control system.^[^ ^11^, ^12^ ^]^

TIA1, an RBP, is a crucial component of SGs. TIA1 plays a key role in regulating cellular stress responses, controlling inflammation, and modulating immune cell functions.^[^ ^13^, ^14^ ^]^ The basis of Welander distal myopathy has been confirmed to be associated with TIA1 dysfunction, with the primary pathological mechanism likely involving changes in the dynamics of SGs.^[^ ^15^ ^]^ Similarly, dysfunctional TIA1 is considered a fundamental factor in the neurodegenerative process of amyotrophic lateral sclerosis and frontotemporal dementia.^[^ ^16^ ^]^ Current research indicates that biological alterations in SGs and RBPs contribute to the neurodegenerative changes in experimental autoimmune encephalomyelitis (EAE), although the specific mechanisms remain unclear.^[^ ^3^ ^]^ Unlike conventional prions and prion‐related proteins, which are generally associated with neurodegenerative disease in animals, TIA1 aggregation is highly regulated and likely serves several critical physiological processes in a positive capacity.^[^ ^17^, ^18^ ^]^ Although previous studies have shown that TIA1 is critical for the dynamics of SGs response to cellular stress, modulating immune cell functions and inflammation, and involving in neurodegeneration, it remains poorly understood that the roles and mechanisms of TIA1‐mediated SGs in MS. In‐depth investigation of TIA1 and the changes in TIA1‐mediated SGs in MS may provide a new therapeutic target for MS treatment.

In this study, we found that the upregulation of TIA1 in the spinal cord neurons of EAE mice, and TIA1 knockout in the CNS alleviated the neuroinflammation, demyelination, and axonal damage, and reduced the neuronal loss in EAE mice. Mechanically, we found that autophagy dysfunction was alleviated, and chronic persistent SGs were reduced in *Tia1* ^Nestin^‐CKO EAE mice through downregulation of IL‐31 RA/PI3K/AKT signaling. These results suggest that TIA1 in the CNS promotes EAE by enhancing the IL‐31RA/PI3K/AKT signaling pathway and providing insights into the mechanisms of neuroinflammation and demyelination in EAE, contributing to the development of new therapeutic approaches for MS.

## 2 Results

### 2.1 TIA1 was Upregulated and Trans‐Localized from the Cell Nucleus to the Cytoplasm in Spinal Cord Neurons of EAE Mice

**Figure 1:**
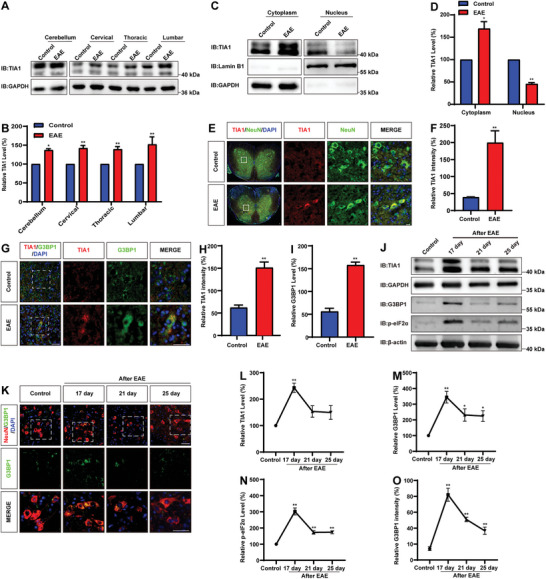
TIA1 was upregulated and trans‐localized from the cell nucleus to the cytoplasm in spinal cord neurons of EAE mice. A) Western blot analysis of TIA1 expression in the cerebellum, cervical, thoracic, and lumbar spinal cords of control and EAE mice. B) Quantitative analysis of the relative TIA1 levels as shown in (A) (normalized to control mice, *n* = 4 blots from 4 mice per group, paired *t*‐test). C) Western blot analysis of TIA1 expression in spinal cord cytoplasm and nucleus of control and EAE mice. D) Quantitative analysis of the relative TIA1 levels as shown in (C) (normalized to control mice, *n* = 5 blots from 5 mice per group, paired *t*‐test). E) Double immunostaining analysis of TIA1 (red) and NeuN (green) in spinal cords of control and EAE mice. F) Quantitative analysis of the intensity of TIA1 as shown in (E) (*n* = 4 sections from 4 mice per group). G) Double immunostaining analysis of TIA1 (red) and G3BP1 (green) in control and EAE mice. H) Quantitative analysis of the intensity of TIA1 as shown in (G) (*n* = 4 sections from 4 mice per group). I) Quantitative analysis of the intensity of G3BP1 as shown in (G) (*n* = 4 sections from 4 mice per group). J) Western blot analysis of TIA1, G3BP1, and p‐eIF2α expression in spinal cords of control and EAE mice at 17, 21, and 25 days after EAE. K) Double immunostaining analysis of G3BP1 (green) and NeuN (red) in control and EAE mice at 17, 21, and 25 days after EAE. L–N) Quantitative analysis of the relative TIA1 levels (L), G3BP1 level (M), and p‐eIF2α levels (N) as shown in (J) (normalized to control mice, *n* = 4 sections from 4 mice per group each group, one‐way ANOVA). O) Quantitative analysis of the intensity of G3BP1 as shown in (K) (*n* = 3 sections from 3 mice per group, one‐way ANOVA). Scale bars, 20 µm. Data were mean ± SEM. Student's *t‐*test unless otherwise indicated, ^*^ *p* < 0.05, ^**^ *p* < 0.01.

To investigate the potential role of TIA1 in MS, we established an EAE model induced by myelin oligodendrocyte glycoprotein (MOG). Interestingly, as shown in **Figure** **1**A,B, TIA1 was significantly upregulated within the spinal cord‐encompassing cervical, thoracic, and lumbar segments as well as in the cerebellum of the EAE mice. Intriguingly, western blot analysis showed a significant increase in the cytoplasmic protein levels of TIA1 in the spinal cord cells of EAE mice, while there was a discernible decrease of TIA1 within the nuclei of these cells (Figure 1C,D). Furthermore, TIA1 was predominantly localized in NeuN^+^ neurons, with a markedly enhanced immunofluorescence intensity of TIA1 in EAE mice (Figure 1E,F).

We next examined whether other related SGs proteins such as G3BP1 underwent similar changes in the CNS as TIA1. As expected, an enhanced immunofluorescence intensity of TIA1 and G3BP1 was observed, demonstrating co‐localization within the spinal cord tissue of EAE mice (Figure 1G–I). We also conducted a time point western blot analysis for TIA1 protein levels in the lumbar spinal cord of EAE mice and found TIA1 protein levels reached their zenith on the 17th day following EAE induction, subsequently exhibiting a gradual decline on the 21st and 25th days (Figure 1J,L). A similar trend was observed in the protein levels of G3BP1 and phosphorylated‐ eIF2α (p‐eIF2α), which also peaked on the 17th day post‐modeling and diminished progressively on the 21st and 25th days (Figure 1J,M,N). Notably, the expression of G3BP1 in neurons was at its highest on the 17th day post‐EAE modeling, with a subsequent gradual decrease over time (Figure 1K,O). These results suggested that TIA1 was upregulated and trans‐localized from the cell nucleus to the cytoplasm in spinal cord neurons of EAE mice, and with dynamic change during EAE.

### 2.2 EAE was Relieved with Later Onset, and Neuroinflammatory Infiltration and Neuronal Loss were Inhibited, while Peripheral Immunity Remained Unaffected in *Tia1* ^Nestin^‐CKO EAE Mice

To further explore the role of TIA1 in EAE, the floxed *Tia1* allele (*Tia1* ^f/f^) mice were crossed with Nestin‐Cre transgenic mice to generate *Tia1* ^Nestin^‐CKO mice, which conditionally ablation of TIA1 specifically in neural stem cells and their derivatives including neurons (Figure S1A, Supporting Information). Indeed, TIA1 was effectively knockout in the spinal cords and brain regions including the cortex, cerebellum, and olfactory bulb of *Tia1* ^Nestin^‐CKO mice (Figure S1B,C, Supporting Information). Surprisingly, there were no significant differences in body weight or number and distribution of neurons in the spinal cord between the *Tia1* ^f/f^ and *Tia1* ^Nestin^‐CKO mice (Figure S1D–K, Supporting Information), which suggested that deletion of TIA1 in the CNS did not affect spinal cord development.

**Figure 2:**
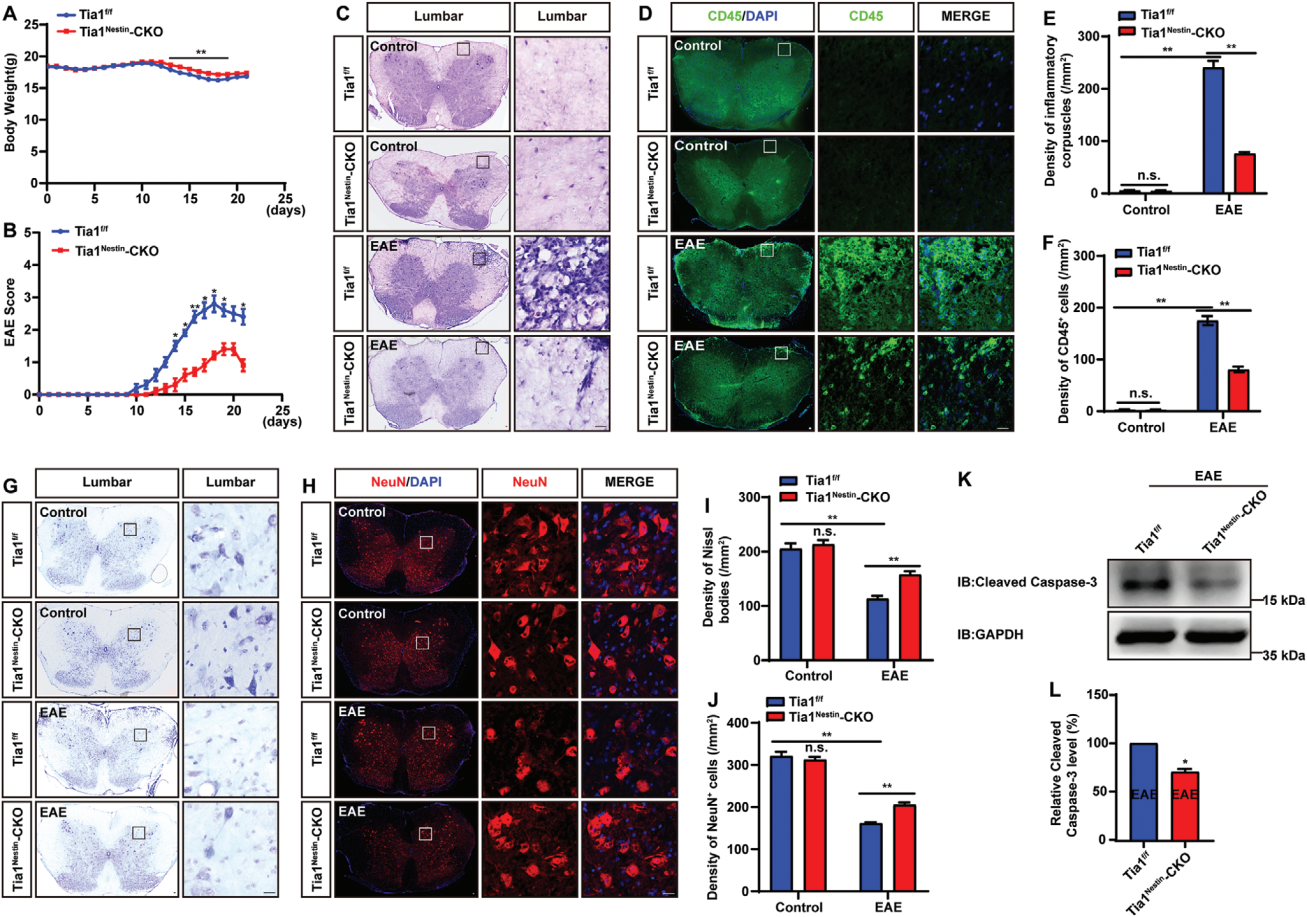
EAE onset was delayed, and the inflammatory infiltration and neuronal loss were alleviated in *Tia1* ^Nestin^‐CKO mice. A,B) The weight (A) and EAE score (B) of *Tia1* ^f/f^ EAE mice and *Tia1* ^Nestin^ *‐CKO* EAE mice ranged from 0 to 21 days after EAE (*n* = 5 mice per group, two‐way ANOVA with Bonferroni's post‐tests). C,D) The typical images of HE staining (C), and CD45^+^ immunostaining (D) in the lumbar spinal cords of *Tia1* ^f/f^ mice and *Tia1* ^Nestin^‐CKO mice, and *Tia1* ^f/f^ EAE mice and *Tia1* ^Nestin^‐CKO EAE mice. E,F) Quantitative analysis of the density of infiltrating cells as shown in (C) (E, *n* = 4 sections from 4 mice per group, two‐way ANOVA), and CD45^+^ cells as shown in (D) (F, *n* = 4 sections from 4 mice per group, two‐way ANOVA). G,H) Typical images of Nissl staining (G) and NeuN immunostaining (H) in the lumbar spinal cords of *Tia1* ^f/f^ mice and *Tia1* ^Nestin^‐CKO mice, and *Tia1* ^f/f^ EAE mice and *Tia1* ^Nestin^‐CKO EAE mice. I,J) Quantitative analysis of the density of Nissl bodies as shown in (G) (I, *n* = 5 sections from 5 mice per group, two‐way ANOVA), and NeuN^+^ cells as shown in (H) (J, *n* = 4 sections from 4 mice per group, two‐way ANOVA). K) Western blot analysis of cleaved caspase‐3 expression in spinal cords of *Tia1* ^f/f^ EAE mice and *Tia1* ^Nestin^‐CKO EAE mice. L) Quantitative analysis of the relative cleaved caspase‐3 levels as shown in (K) (normalized to control mice, *n* = 3 blots from 3 mice per group, paired *t‐*test). Scale bars, 20 µm. Data were mean ± SEM. n.s., not significant. ^*^ *p* < 0.05, ^**^ *p* < 0.01.

Next, female mice from *Tia1* ^f/f^ and *Tia1* ^Nestin^‐CKO groups weighing 15–20 g were subjected to EAE induction utilizing MOG35‐55 and pertussis toxin. Compared to *Tia1* ^f/f^ EAE mice, *Tia1* ^Nestin^‐CKO EAE mice exhibited less weight loss (**Figure** **2**A). In addition, *Tia1* ^Nestin^‐CKO EAE mice showed a delayed onset of the initial symptomatic manifestation based on tail paralysis and a substantially reduced EAE score (Figure 2B). The results of HE staining showed that there were no significant differences in the density of infiltrating cells in *Tia1* ^f/f^ mice and *Tia1* ^Nestin^‐CKO mice. However, after EAE induction, *Tia1* ^Nestin^‐CKO EAE mice showed a significantly lower density of infiltrating cells (Figure 2C,E). In addition, there were no differences in the density of CD45^+^ cells between *Tia1* ^f/f^ mice and *Tia1* ^Nestin^‐CKO mice (Figure 2D,F). However, *Tia1* ^Nestin^‐CKO EAE mice had a significantly lower density of CD45^+^ cells in their spinal cord. The results of RT‐PCR further confirmed that TIA1 deletion decreased the expression of pro‐inflammatory factors such as IL1, IL6, TNFα, and INFγ, while simultaneously enhancing the expression of immune‐regulatory factors CD206 and TGFβ (Figure S2A–F, Supporting Information).

FACS analysis revealed a marked increase in the population of T cells (CD45^+^, CD4^+^), myeloid cells, and macrophage cells (CD45^+^ and CD11b^+^) in the spleen of EAE mice in comparison to the control mice (Figure S3A–C, Supporting Information). However, there were no significant differences in these immune cells between *Tia1* ^f/f^ EAE mice and *Tia1* ^Nestin^‐CKO EAE mice. In terms of the number of CD4^+^ T cells, the percentage of Th1 and Th17 cells was significantly higher in the spleen of EAE mice compared to the control mice (Figure S3D–F, Supporting Information). Similarly, there was no significant difference in the percentage of Th1 and Th17 cells between *Tia1* ^f/f^ EAE mice and *Tia1* ^Nestin^‐CKO EAE mice. Taken together, these results suggested that TIA1 deletion in the CNS did not affect T cell populations within the spleen in EAE mice.

Neuronal loss is one of the core pathological features of MS and EAE.^[^ ^19^ ^]^ As expected, Nissl staining analysis revealed a marked reduction in the density of Nissl bodies within the spinal cord tissue of EAE mice. Interestingly, the reduction of Nissl bodies was inhibited in *Tia1* ^Nestin^‐CKO EAE mice (Figure 2G,I). Similarly, immunostaining showed that the loss of NeuN^+^ cells was also significantly inhibited in the lumbar spinal cord of *Tia1* ^Nestin^‐CKO EAE mice (Figure 2H,J). Consistent with these findings, western blot analysis also showed that the levels of pro‐apoptotic proteins, specifically cleaved caspase‐3, were also significantly decreased in the spinal cord tissue of *Tia1* ^Nestin^‐CKO EAE mice (Figure 2K,L). Collectively, these results suggested that in *Tia1* ^Nestin^‐CKO mice, the progression of EAE was mitigated, characterized by a delayed onset, reduced inflammatory infiltration, and fewer neuron loss, thereby indicating a potential protective role of TIA1 knockout in the pathophysiology of EAE.

### 2.3 The Activation of Astrocytes and Microglia was Suppressed, and the Proliferation of Astrocytes and Microglia was Reduced in the Spinal Cords of *Tia1* ^Nestin^‐CKO EAE Mice

**Figure 3:**
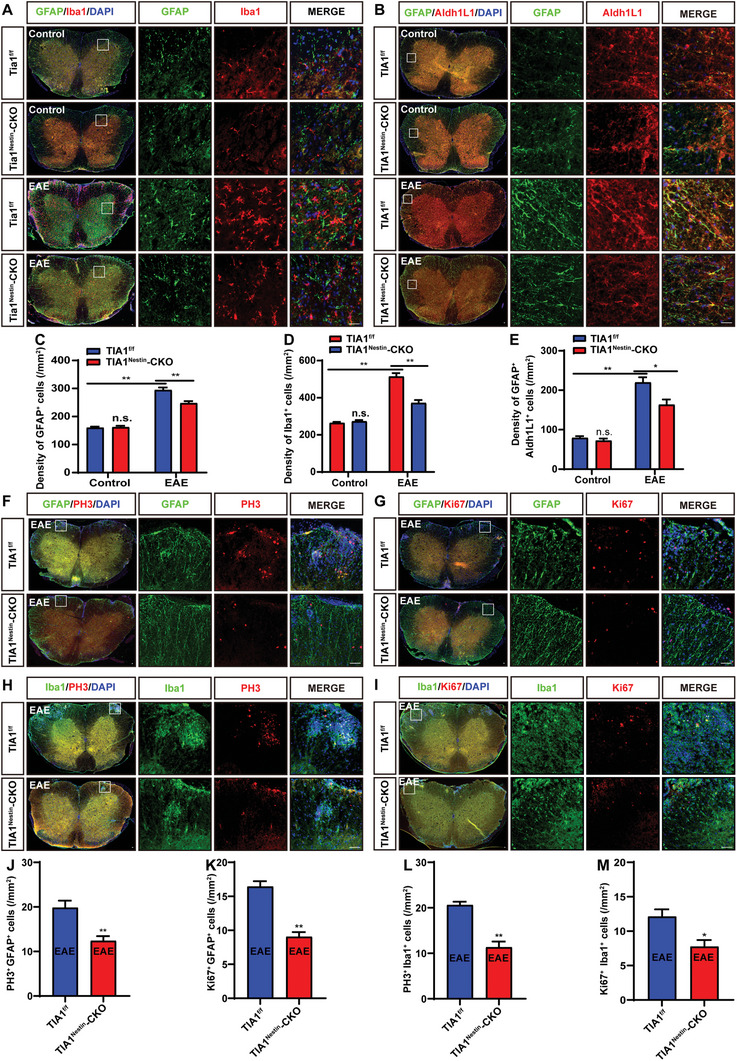
The activation and proliferation of astrocytes and microglia were suppressed in the spinal cords of *Tia1* ^Nestin^‐CKO EAE mice. A,B) Double immunostaining of GFAP (green) and Iba1 (red) (A), and GFAP (green) and Aldh1L1 (red) (B) in lumbar spinal cords of *Tia1* ^f/f^ mice and *Tia1* ^Nestin^‐CKO mice, and *Tia1* ^f/f^ EAE mice and *Tia1* ^Nestin^‐CKO EAE mice. C–E) Quantitative analysis of the density of GFAP^+^ (C) and Iba1^+^ (D) as shown in (A) (*n* = 5 sections from 5 mice per group, two‐way ANOVA), and GFAP^+^/Aldh1L1^+^ (E) as shown in (B) (*n* = 4 sections from 4 mice per group, two‐way ANOVA). F–I) Double immunostaining analysis of GFAP (green) and PH3 (red) (F), GFAP (green) and Ki67 (red) (G), Iba1 (green) and Ki67 (red) (H) and Iba1 (green) and Ki67 (red)(I) in lumbar spinal cords of *Tia1* ^f/f^ mice and *Tia1* ^Nestin^‐CKO mice, and *Tia1* ^f/f^ EAE mice and *Tia1* ^Nestin^‐CKO EAE mice. J–M) Quantitative analysis of the density of PH3^+^/GFAP^+^ as shown in (F) (J, *n* = 4 sections from 4 mice per group), the density of Ki67^+^/GFAP^+^ as shown in (G) (K, *n* = 4 sections from 4 mice per group), the density of PH3^+^/Iba1^+^ as shown in (H) (L, *n* = 4 sections from 4 mice per group), and the density of Ki67^+^/Iba1^+^ as shown in (I) (M, *n* = 4 sections from 4 mice per group). Scale bars, 20 µm. Data were mean ± SEM. Student's *t*‐test unless otherwise indicated, n.s., not significant. ^*^ *p* < 0.05, ^**^p < 0.01.

In EAE and MS, the glial cell response promotes the occurrence and progression of inflammation and neural damage.^[^ ^2^ ^]^ Therefore, we investigated the impact of CNS‐specific TIA1 knockout on glial cell responses. Double immunostaining revealed no notable significant difference in the density of GFAP^+^ astrocytes and Iba1^+^ microglia between the *Tia1* ^f/f^ and *Tia1* ^Nestin^‐CKO mice (**Figure** **3**A,C,D), however, after EAE induction, marked activation of astrocytes and microglia was observed, characterized by cell enlargement and increased GFAP and Iba1 expression. Notably, these cells also exhibited a significant increase in density. However, the density of GFAP^+^ astrocytes and Iba1^+^ microglia were significantly decreased in the spinal cord of *Tia1* ^Nestin^‐CKO EAE mice (Figure 3A,C,D). Furthermore, double immunostaining of GFAP and ALDH1L1 also showed that astrocytes were significantly more activated in Tia1^f/f^ EAE mice, whereas this activation was comparatively mitigated in *Tia1* ^Nestin^‐CKO EAE mice (Figure 3B,E). Additionally, a decrease in the percentages of PH3^+^ and Ki67^+^ astrocytes relative to the total GFAP^+^ astrocyte population was found in *Tia1* ^Nestin^‐CKO EAE mice (Figure 3F,G,J,K). Similarly, the proliferation rates of microglia in the spinal cord of *Tia1* ^Nestin^‐CKO EAE mice were also lower than those in *Tia1* ^f/f^ EAE mice (Figure 3H,I,L,M). These results collectively suggested that the glial cell response, encompassing the activation and proliferation of astrocytes and microglia, as well as the expression levels of GFAP and ALDH1L1, was attenuated in *Tia1* ^Nestin^‐CKO EAE mice.

### 2.4 The Demyelination and Axonal Injury were Alleviated in the Spinal Cords of *Tia1* ^Nestin^‐CKO EAE Mice

**Figure 4:**
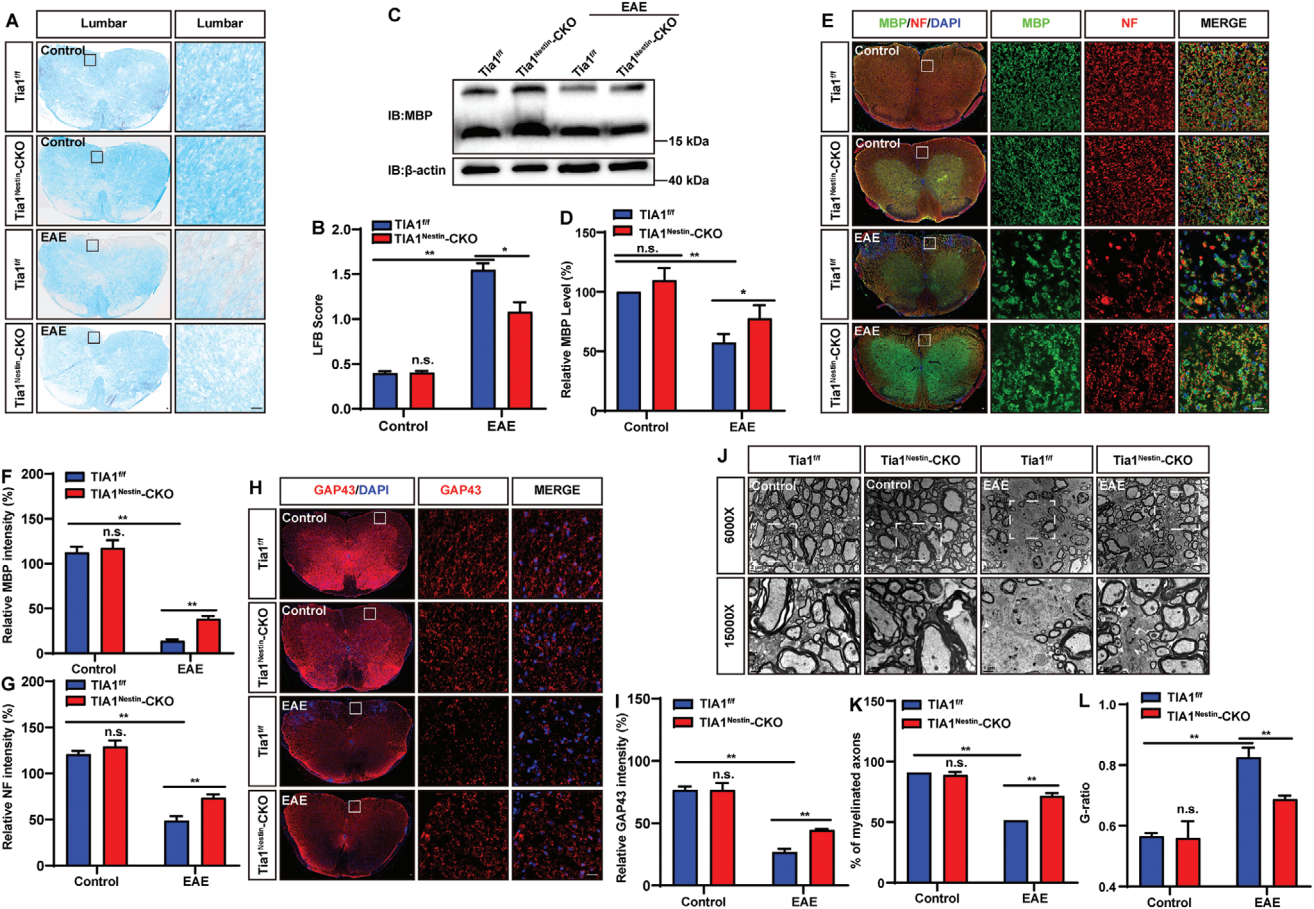
The demyelination and axonal injury were alleviated in the spinal cords of *Tia1* ^Nestin^‐CKO EAE mice. A) The typical LFB staining of lumbar spinal cords in *Tia1* ^f/f^ mice and *Tia1* ^Nestin^‐CKO mice, and *Tia1* ^f/f^ EAE mice and *Tia1* ^Nestin^‐CKO EAE mice. B) Quantitative analysis of the demyelination score (*n* = 4 sections from 4 mice per group, two‐way ANOVA). C) Western blot analysis of MBP expression in spinal cords of *Tia1* ^f/f^ mice and *Tia1* ^Nestin^‐CKO mice, *Tia1* ^f/f^ EAE mice, and *Tia1* ^Nestin^‐CKO EAE mice. The higher molecular weight band is likely the 21.5 kDa isoform, whereas the lower molecular weight bands could correspond to the 18.5 and 17.2 kDa isoforms. D) Quantitative analysis of the relative MBP level as shown in (C) (normalized to *Tia1* ^f/f^ mice, *n* = 4 blots from 4 mice per group, two‐way ANOVA). E,H) Immunostaining of MBP (green) and NF (red)(E), and GAP43 (red) (H) in spinal cords of *Tia1* ^f/f^ mice and *Tia1* ^Nestin^‐CKO mice, and *Tia1* ^f/f^ EAE mice and *Tia1* ^Nestin^‐CKO EAE mice. F–G) Quantitative analysis of the relative expression of MBP (F, *n* = 5 sections from 5 mice per group, two‐way ANOVA) and NF (G, *n *= 4 sections from 4 mice per group, two‐way ANOVA) as shown in (E). I) Quantitative analysis of the relative GAP43 expression (*n* = 5 sections from 5 mice per group, two‐way ANOVA) as shown in (H). J) The typical electron microscopic images of lumbar spinal cords in *Tia1* ^f/f^ mice and *Tia1* ^Nestin^‐CKO mice, and *Tia1* ^f/f^ EAE mice and *Tia1* ^Nestin^‐CKO EAE mice. Scale bars, 2 µm (low power image) and 1 µm (high power image). K–L) Quantitative analysis of the percentage of myelinated axons (K) and G‐ratio (L) as shown in (J) (*n* = 3 per group, two‐way ANOVA). Scale bars, 20 µm. Data were mean ± SEM. n.s., not significant. ^*^ *p* < 0.05, ^**^p < 0.01.

Axonal damage and demyelination represent fundamental pathological characteristics of MS/EAE.^[^ ^20^ ^]^ LFB staining was first performed to evaluate the effects of TIA1 knockout on myelin loss. The results revealed significant mitigation of the demyelination phenotype in *Tia1* ^Nestin^‐CKO EAE mice (**Figure** **4**A,B). Western blot analysis further showed that TIA1 knockout in the CNS attenuated the reduction of Myelin Basic Protein (MBP) in the EAE mice (Figure 4C,D). These results were corroborated by immunostaining, which unveiled notable discrepancies in the fluorescent intensity of neurofilament heavy polypeptide (NF) and MBP between the control and EAE mice. Specifically, a significant decrease in the fluorescent intensity of both MBP and NF was observed in the spinal cord of EAE mice. Nonetheless, compared to *Tia1* ^f/f^ EAE mice, *Tia1* ^Nestin^‐CKO EAE mice exhibited significant inhibition of the reduction in the fluorescent intensity of both MBP and NF (Figure 4E–G).

Additionally, the immunostaining intensity of Growth Associated Protein 43 (GAP43) was markedly elevated in the *Tia1* ^Nestin^‐CKO EAE mice (Figure 4H,I). Consistent with these findings, electron microscopy showed a substantial amelioration of the demyelination phenotype in *Tia1* ^Nestin^‐CKO EAE mice compared to *Tia1* ^f/f^ EAE mice (Figure 4J–L). Collectively, TIA1 knockout in the CNS effectively mitigated the axonal damage and demyelination in the spinal cord of EAE mice.

### 2.5 The SGs were Decreased and Autophagy was Enhanced in the Spinal Cords of *Tia1* ^Nestin^‐CKO EAE Mice

**Figure 5:**
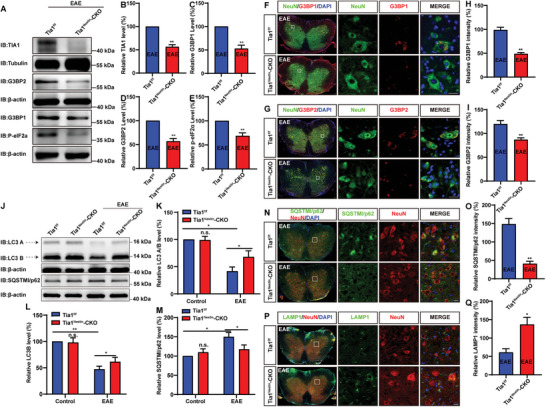
The SGs were decreased and autophagy was enhanced in the spinal cords of *Tia1^Nestin^*‐CKO EAE mice. A) Western blot analysis of TIA1, G3BP1, G3BP2, and p‐eIF2α expression in spinal cords of *Tia1* ^f/f^ EAE mice and *Tia1* ^Nestin^‐CKO EAE mice. B–E) Quantitative analysis of the relative TIA1 (B), G3BP1 (C), G3BP2 (D), and p‐eIF2α levels (E) as shown in (A) (normalized to *Tia1* ^f/f^ EAE mice, *n* = 5 blots from 5 mice per group, paired *t*‐test). F,G) Double immunostaining of NeuN (green) and G3BP1 (red) (F), and NeuN (green) and G3BP2 (red) (G) in spinal cords of *Tia1* ^f/f^ EAE mice and *Tia1* ^Nestin^‐CKO EAE mice. H,I) Quantitative analysis of the relative G3BP1 expression as shown in (F) (H, *n* = 5 sections from 5 mice per group), and the relative G3BP2 expression as shown in (G) (I, *n* = 5 sections from 5 mice per group). J) Western blot analysis of LC3 and SQSTMI/p62 expression in spinal cords of *Tia1* ^f/f^ mice and *Tia1* ^Nestin^‐CKO mice, and *Tia1* ^f/f^ EAE mice and *Tia1* ^Nestin^‐CKO EAE mice. The band of higher molecular weight may be 16 kDa isoform, while that of lower molecular weight may represent 14 kDa isoform. K–M) Quantitative analysis of the relative LC3 A/B level (K) (normalized to *Tia1* ^f/f^ mice, *n* = 3 blots from 3 mice per group), LC3 B (L) (normalized to *Tia1* ^f/f^ mice, *n* = 4 blots from 4 mice per group, two‐way ANOVA) and SQSTMI/p62 (M) (normalized to *Tia1* ^f/f^ mice, *n *= 3 blots from 3 mice per group, two‐way ANOVA) as shown in (J). N,P) Double immunostaining of SQSTMI/p62 (green) and NeuN (red) (N), LAMP1 (green) and NeuN (red) (P) in spinal cords of *Tia1* ^f/f^ EAE mice and *Tia1* ^Nestin^‐CKO EAE mice. O,Q) Quantitative analysis of the relative SQSTMI/p62 expression as shown in (N) (O, *n* = 4 sections from 4 mice per group), and the relative LAMP1 expression as shown in (P) (Q, *n* = 4 sections from 4 mice per group). Scale bars, 20 µm. Data were mean ± SEM. Student's *t‐*test unless otherwise indicated, n.s., not significant. ^*^ *p* < 0.05, ^**^ *p* < 0.01.

Next, we examined the effects of TIA1 knockout on SGs in EAE mice. As expected, as shown in **Figure** **5**A–E, the expression of TIA1, G3BP1, G3BP2, and p‐eIF2α were markedly reduced in *Tia1* ^Nestin^‐CKO EAE mice (Figure 5A–E). Furthermore, as shown in Figure 5F–I, the expression of G3BP1 and G3BP2 was significantly reduced in the spinal cord neurons of *Tia1* ^Nestin^‐CKO EAE mice. These findings demonstrated that the knockout of TIA1 in neurons inhibited SGs.

Autophagy is considered an effective pathway for the clearance of SGs, which helps to maintain cellular homeostasis.^[^ ^9^, ^21^, ^22^ ^]^ Interestingly, the result of the western blot showed a significant reduction of LC3B, a key autophagy marker, in the spinal cord tissue of EAE mice. However, LC3B showed a notable increased in *Tia1* ^Nestin^‐CKO EAE mice (Figure 5J,L). SQSTM1/p62 serves as a crucial adaptor protein for selective autophagy, functioning as a receptor in the removal of ubiquitinated proteins. Upon lysosomal degradation, p62 bound to the substrate is broken down by proteases.^[^ ^23^ ^]^ Therefore, SQSTM1/p62 is usually considered a sign of reduced autophagic activity. As expected, both western blot and immunostaining showed that the reduction of SQSTM1/p62 was also significantly inhibited in *Tia1* ^Nestin^‐CKO EAE mice (Figure 5J,M–O). LAMP1 serves as a vital membrane protein found in both autophagosomes and lysosomes, with its expression level indicating the cell's autophagic capability. It can also be utilized to quantitatively assess variations in autophagy levels and appraise the functional state of the autophagy pathway.^[^ ^24^ ^]^ Interestingly, the reduction of LAMP1 was also inhibited in the spinal cord neurons of *Tia1* ^Nestin^‐CKO EAE mice (Figure 5P,Q). In summary, these results suggested that the SGs were reduced and autophagy was enhanced in spinal cord neurons of *Tia1* ^Nestin^‐CKO EAE mice.

### 2.6 The Immune Process was Regulated, and the Expression of IL‐31RA was Decreased in *Tia1* ^Nestin^‐CKO EAE Mice

**Figure 6:**
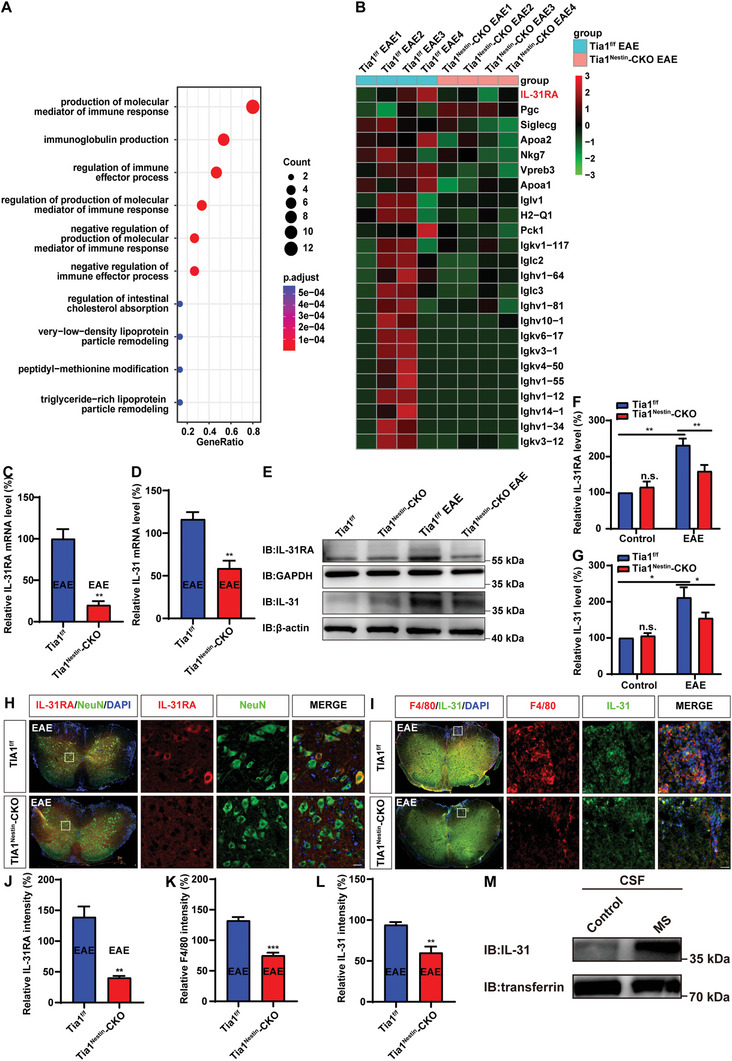
IL‐31RA was reduced in spinal cord neurons in *Tia1* ^Nestin^‐CKO EAE mice. A) GO analysis of RNA‐seq in spinal cord tissue of *Tia1* ^f/f^ EAE mice and *Tia1* ^Nestin^‐CKO EAE mice (*n* = 4 mice per group). B) The heatmap of differential mRNAs in spinal cords of *Tia1* ^f/f^ EAE mice and *Tia1* ^Nestin^ *‐CKO* EAE mice (*n* = 4 mice per group) based on RNA‐seq. C,D) Analysis of qPCR showed the relative mRNA level of IL‐31RA (C, *n* = 6 mice per group) and IL‐31 (D, *n* = 4 mice per group) in spinal cords of *Tia1* ^f/f^ EAE mice and *Tia1* ^Nestin^‐CKO EAE mice (*n* = 6 mice per group). E) Western blot analysis of the IL‐31RA and IL‐31 expression in spinal cords of *Tia1* ^f/f^ mice and *Tia1* ^Nestin^‐CKO mice, *Tia1* ^f/f^ EAE mice and *Tia1* ^Nestin^‐CKO EAE mice. F,G) Quantitative analysis of the relative IL‐31RA levels (F, normalized to *Tia1* ^f/f^ mice, *n* = 4 blots from 4 mice per group, two‐way ANOVA), and the relative IL‐31 levels (G, normalized to *Tia1* ^f/f^ mice, *n* = 5 blots from 5 mice per group, two‐way ANOVA) as shown in (E). H,I) Double immunostaining of IL‐31RA (red) and NeuN (green) (H), and IL‐31 (green) and F4/80 (red) (I) in spinal cords of *Tia1* ^f/f^ EAE mice and *Tia1* ^Nestin^‐CKO EAE mice. J) Quantitative analysis of the relative IL‐31RA expression as shown in (H) (*n* = 4 sections from 4 mice per group). K,L) Quantitative analysis of the relative F4/80 expression (K, *n* = 4 sections from 4 mice per group) and IL‐31 expression (L, *n* = 4 sections from 4 mice per group) as shown in (I). M) Western blot analysis of IL‐31 expression in CSF of normal person and MS patients. Scale bars, 20 µm. Data were mean ± SEM. Student's *t‐*test unless otherwise indicated, n.s., not significant. ^*^ *p* < 0.05, ^**^ *p* < 0.01, ^***^ *p* < 0.001.

RNA‐sequencing was employed to elucidate how TIA1 knockout alleviated the deficits of EAE mice, followed by Gene Ontology (GO) analysis to dissect the biological processes potentially implicated. The Biological Process (BP) category of the GO analysis indicated that TIA1 knockout in CNS predominantly influenced the production of molecular mediators of immune response, immunoglobulin production, and other immune‐related BPs. Notably, the majority of these BPs are recognized for their anti‐inflammatory effects or positive roles in immune regulation (**Figure** **6**A). Intriguingly, a downregulation in the expression of pro‐inflammatory cytokine genes and an upregulation of anti‐inflammatory cytokine genes were observed in the *Tia1* ^Nestin^‐CKO EAE mice compared to the *Tia1* ^f/f^ EAE mice, with a significant decrease in the IL‐31RA gene expression being particularly noteworthy (Figure 6B). Western blot and q‐PCR analyses further confirmed the decrease of IL‐31 and IL‐31RA in *Tia1* ^Nestin^‐CKO EAE mice (Figure 6C–G). Additionally, IL‐31RA was predominantly expressed in NeuN^+^ neurons in the lumbar spinal cord, and TIA1 knockout in the CNS effectively suppressed the upregulation of IL‐31RA in EAE mice (Figure 6H,J). Moreover, a decrease in macrophages and a concurrent reduction in IL‐31 secretion were observed in the spinal cord of *Tia1* ^Nestin^‐CKO EAE mice (Figure 6I,K,L). Western Blot of the patient's cerebrospinal fluid showed that the expression of IL31 in the cerebrospinal fluid of MS patients were significantly upregulated (Figure 6M). Taken together, these results suggested that TIA1 knockout in the CNS modulated the immune‐related BPs, particularly decreasing the gene expression of IL‐31RA.

### 2.7 The IL31‐RA/PI3K/AKT/NF‐κB Signaling Pathway was Inhibited in *Tia1* ^Nestin^‐CKO EAE Mice

**Figure 7:**
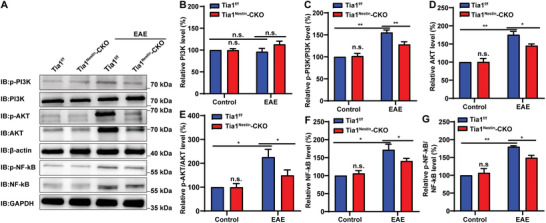
The IL31‐Ra/PI3K/AKT/NF‐κB signaling pathway was inhibited in *Tia1* ^Nestin^‐CKO EAE mice. A) Western blot analysis of p‐PI3K, PI3K, p‐AKT, AKT, p‐NF‐κB and NF‐κB expression in spinal cords of *Tia1* ^f/f^ mice and *Tia1* ^Nestin^‐CKO mice, *Tia1* ^f/f^ EAE mice and *Tia1* ^Nestin^‐CKO EAE mice. B–G) Quantitative analysis of the relative PI3K (B), p‐PI3K/PI3K (C), AKT (D), p‐AKT/AKT (E), NF‐κB (F), and p‐NF‐κB/NF‐κB (G) levels as shown in (A) (normalized to *Tia1* ^f/f^ mice, *n* = 4 blots from 4 mice per group, two‐way ANOVA). Data were mean ± SEM, n.s., not significant. ^*^ *p* < 0.05, ^**^ *p* < 0.01.

When IL‐31 activates the IL‐31RA/OSMR complex, PI3K can be phosphorylated, leading to a significant increase in subsequent AKT phosphorylation.^[^ ^25^ ^]^ Therefore, we next examined whether the PI3K/AKT signaling pathway was inhibited in *Tia1* ^Nestin^‐CKO EAE mice. Indeed, the results of the western blot showed that phosphorylated PI3K (p‐PI3K) and AKT (p‐AKT), as well as the total protein levels of AKT, were increased in the spinal cord tissue of *Tia1* ^f/f^ EAE mice compared to the control group, while the total protein levels of PI3K remained relatively unchanged. However, the increase of the p‐PI3K and p‐AKT was significantly inhibited in *Tia1* ^Nestin^‐CKO EAE mice (**Figure** **7**A–E). Meanwhile, the p‐NF‐κB was upregulated in *Tia1* ^f/f^ EAE mice, while the upregulation of p‐NF‐κB was also significantly inhibited in *Tia1* ^Nestin^‐CKO EAE mice (Figure 7A,F,G). These findings showed that the neuroinflammatory response was inhibited in *Tia1* ^Nestin^‐CKO EAE mice by downregulating the PI3K/AKT/NF‐κB pathway.

### 2.8 PI3K Inhibitor Relieved the Neuroinflammation and Demyelination Deficits in *Tia1* ^Nestin^‐CKO EAE Mice

**Figure 8:**
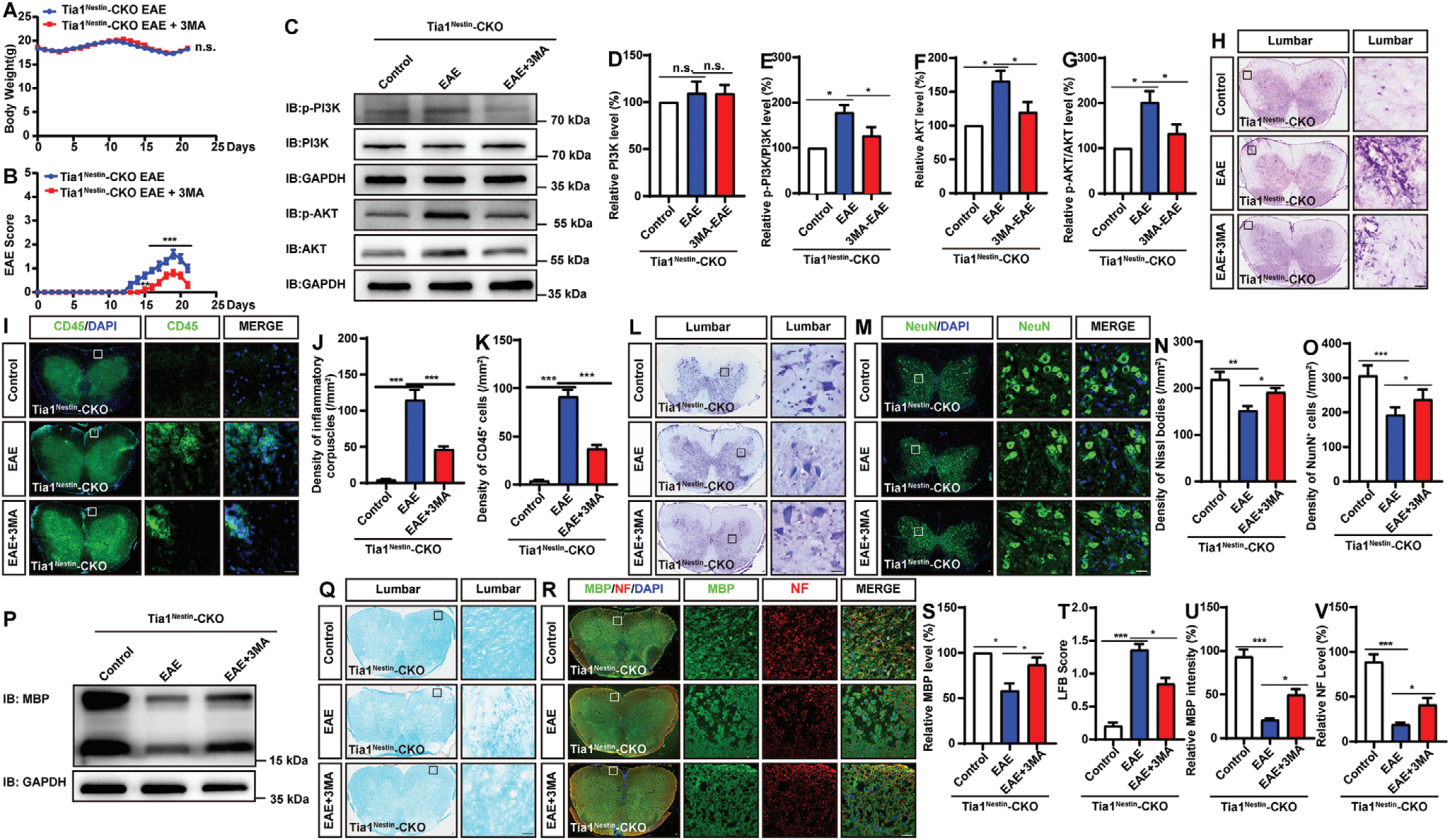
The neuroinflammation and demyelination deficits were relieved by PI3K inhibitor in *Tia1* ^Nestin^‐CKO EAE mice. A,B) The weight (A) and EAE score (B) of *Tia1* ^Nestin^‐CKO mice, *Tia1* ^Nestin^‐CKO EAE mice, and *Tia1* ^Nestin^‐CKO EAE mice treated with 3‐MA ranged from 0 to 21 days after EAE (*n* = 5 mice per group, two‐way ANOVA with Bonferroni's post‐tests). C) Western blot analysis of p‐PI3K, PI3K, p‐AKT, and AKT expression in spinal cords of *Tia1* ^Nestin^‐CKO mice, *Tia1* ^Nestin^‐CKO EAE mice, and *Tia1* ^Nestin^‐CKO EAE mice treated with 3‐MA. D–G) Quantitative analysis of the relative PI3K (D), p‐PI3K/PI3K (E), AKT (F), and p‐AKT/AKT (G) levels as shown in (C) (normalized to *Tia1* ^Nestin^‐CKO mice, *n* = 4 blots from 4 mice per group, paired *t‐*test). H,I) The typical images of HE staining (H) and CD45^+^ immunostaining (I) in the lumbar spinal cords of *Tia1* ^Nestin^‐CKO mice, *Tia1* ^Nestin^‐CKO EAE mice and 3‐MA treated *Tia1* ^Nestin^‐CKO EAE mice. J,K) Quantitative analysis of the density of infiltrating cells as shown in (H) (J, *n* = 5 sections from 5 mice per group), and the density of CD45^+^ cells as shown in (I) (K, *n* = 5 sections from 5 mice each group). L,M) Typical images of Nissl staining (L) and NeuN immunostaining (M) in the lumbar spinal cords of *Tia1* ^Nestin^‐CKO mice, *Tia1* ^Nestin^‐CKO EAE mice and 3‐MA treated *Tia1* ^Nestin^‐CKO EAE mice. N,O) Quantitative analysis of the density of Nissl bodies as shown in (L) (N, *n* = 5 sections from 5 mice per group), and the density of NeuN^+^ cells as shown in (M) (O, *n* = 5 sections from 5 mice per group). P) Western blot analysis of MBP expression in spinal cords of *Tia1* ^Nestin^‐CKO mice, *Tia1* ^Nestin^‐CKO EAE mice and 3‐MA treated *Tia1* ^Nestin^‐CKO EAE mice. Q,R) The typical LFB staining (Q), and double immunostaining of MBP (green) and NF (red) (R) in lumbar spinal cords of *Tia1* ^Nestin^‐CKO mice, *Tia1* ^Nestin^‐CKO EAE mice and 3‐MA treated *Tia1* ^Nestin^‐CKO EAE mice. S–V) Quantitative analysis of the relative MBP levels as shown in (P) (S, normalized to *Tia1* ^Nestin^‐CKO mice, *n* = 4 blots from 4 mice per group, paired *t*‐test), the demyelination score as shown in (Q) (T, *n *= 4 sections from 4 mice per group, two‐way ANOVA with Bonferroni's post‐tests), and the relative expression of MBP (U) and NF (V) as shown in (R) (*n *= 4 sections from 4 mice per group). Scale bars, 20 µm. Data were mean ± SEM. Student's *t*‐test unless otherwise indicated, n.s., not significant. ^*^ *p* < 0.05, ^**^ *p* < 0.01, ^***^ *p* < 0.001.

We next examined whether the deficits in *Tia1* ^Nestin^‐CKO EAE mice were mediated by the downregulation of the IL31‐RA/PI3K/AKT/NF‐κB signaling pathway. 3‐methyladenine (3‐MA), a specific PI3K inhibitor, was utilized to treat EAE in *Tia1* ^Nestin^‐CKO mice. Following the administration of 3‐MA, there was no significant change in the body weight of the treated EAE mice (**Figure** **8**A). However, a notable decrease in the EAE score was observed in the *Tia1* ^Nestin^‐CKO EAE mice following 3‐MA treatment (Figure 8B). Additionally, the p‐PI3K and p‐AKT were significantly reduced post‐injection of 3‐MA in these mice (Figure 8C–G). Furthermore, the administration of 3‐MA led to an increase in the density of NeuN^+^ cells and a concomitant decrease in the density of CD45^+^ cells in *Tia1* ^Nestin^‐CKO EAE mice (Figure 8H–O). Interestingly, the intensities of NF and MBP were markedly increased in *Tia1* ^Nestin^‐CKO EAE mice (Figure 8R–V). These findings collectively suggested that TIA1 knockout in the CNS alleviated the neuroinflammation and the demyelination deficits by downregulating the PI3K/AKT/NF‐κB signaling pathway in EAE mice.

### 2.9 TIA1 Knockdown Reduces the Formation of Stress Granules In Vitro Induced by Sodium Arsenite

Sodium arsenite (NaAsO_2_) is a commonly used stress inducer in cell culture, known to trigger the formation of G3BP1^+^ SGs.^[^ ^26^ ^]^ To investigate the role of TIA1‐mediated SGs in neurons, we isolated primary neurons from fetal mice and induced stress granule formation using sodium arsenite. Following sodium arsenite treatment, the levels of TIA1, G3BP1, and p‐eIF2α were significantly elevated in primary neurons (Figure S4A–D, Supporting Information). Furthermore, compared to Tia1^‐/‐^, G3BP1^+^ SGs were significantly reduced in Tia1^+/+^ neurons (Figure S4G–I, Supporting Information).

We further explored the signaling pathway changes mediated by TIA1 in N2a cells in vitro. After sodium arsenite treatment, TIA1 knockdown in N2a cells was accompanied by a decrease in G3BP1^+^ SGs, while the expression of LC3 and LAMP1 was increased (Figure S5C,K,L, Supporting Information). The knockdown of TIA1 likely enhanced autophagy, increasing lysosomal content and thereby reducing SGs. We then investigated the cause of enhanced autophagy. Following TIA1 knockdown, the phosphorylation of PI3K and AKT in N2a cells was also downregulated, which may contribute to the enhancement of autophagy (Figure S5C, Supporting Information), consistent with our in vivo data (as seen in Figure 7).

Oxidative stress is considered one of the key mechanisms underlying the pathogenesis of MS and is known to promote the formation of SGs. To investigate whether the enhanced autophagy induced by TIA1 knockdown affects ROS levels, we measured ROS levels in sodium arsenite‐treated N2a cells. Interestingly, after TIA1 knockdown, ROS levels were significantly lower in the cells compared to the control group, further confirming our findings at the protein level (Figure S5J, Supporting Information).

Next, we verified that the reduction of SGs was indeed caused by the enhancement of autophagy. N2a cells were treated with sodium arsenite and chloroquine (CQ, a common lysosomal inhibitor). Compared to cells without CQ treatment, CQ‐treated cells exhibited an increased number of SGs and reduced LAMP1 expression, indicating that CQ inhibits the degradation of SGs by blocking autophagic lysosomal activity. After TIA1 knockdown, this inhibition was relieved (Figure S5M,N, Supporting Information).

## 3 Discussion

**Scheme 1:**
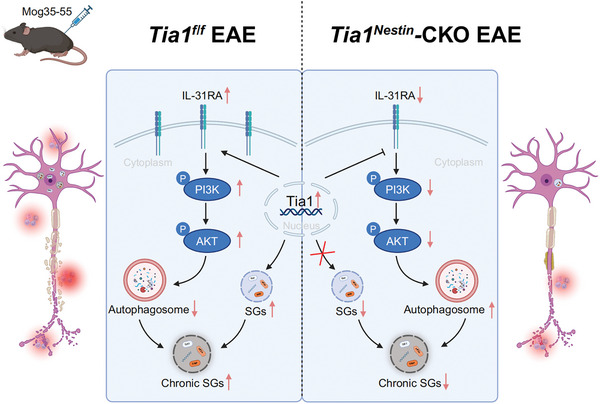
A working model of neuronal TIA1's function in EAE mice. In EAE mice, TIA1 is translocated from the nucleus to the cytoplasm, facilitating SGs formation in neurons, inciting CNS inflammatory infiltration, and precipitating axonal demyelination. TIA1 knockout in the CNS of EAE mice yields a notable diminution in neuronal SGs, mitigates CNS inflammatory infiltration, and curtails axonal demyelination. Mechanistically, CNS‐specific TIA1 knockout engenders a reduction in IL‐31RA expression within spinal cord neurons, triggering a cascade that includes the downregulation of the PI3K/AKT signaling pathway, remediation of autophagy impairments, enhanced degradation of SGs, and a decrease in the chronic accumulation of SGs. These effects collectively contribute to alleviating neuroinflammation and myelin degradation associated with EAE. Created using Biorender.com.

In the present study, utilizing the preclinical EAE mouse model, we provided new evidence (**Scheme** **1**) that TIA1‐mediated IL‐31RA dysregulation was associated with MS pathology. Our studies identified an upregulation of TIA1 expression in neurons, accompanied by a significant accumulation of chronic persistent SGs. Importantly, TIA1 knockout led to significant mitigation of clinical severity without noticeably affecting growth and development in the EAE mice. TIA1 knockout mice exhibited significantly reduced neuroinflammation and demyelination. Furthermore, we found that TIA1 knockout drastically lowered IL‐31RA expression and inhibited the PI3K/AKT signaling pathway in EAE mice. This inhibition not only alleviated the dysfunction of autophagy, thereby reducing the accumulation of chronic persistent SGs, but also suppressed inflammation by inhibiting the NF‐κB (Scheme). Similarly, TIA1 knockdown in N2a cells enhances autophagy and reduces SGs accumulation by inhibiting the PI3K/AKT signaling pathway (Figure S5, Supporting Information).

Studies from Alzheimer's disease experimental and Amyotrophic lateral sclerosis animal models have established a correlation between cytoplasmic deposition of RBPs or SGs and disease severity.^[^ ^16^, ^27^ ^]^ Interestingly, consistent with these previous studies, we observed a similar association in EAE animals, where increased cytoplasmic mis‐localization of TIA1 and SGs correlated with higher clinical scores at the time of euthanasia (Figure 1J–O). TIA1 knockout alleviated the deposition, and EAE was relieved with later onset, less inflammatory infiltration, and fewer neuron loss in *Tia1* ^N^ ^e^ ^s^ ^t^ ^i^ ^n^‐CKO EAE mice (Figure 2).

TIA1 protein has been identified as a key regulatory factor of SGs. Traditionally, SG formation is initiated by stress‐induced p‐e‐IF2α, which requires the self‐aggregation of TIA1. Due to the p‐e‐IF2α, the ternary complex, which connects initiator e‐IF2α‐GTP‐RNAMet with the pre‐initiation complex, may be prevented from forming, thus inhibiting the productive translational initiation.^[^ ^28^ ^]^ Under stress conditions, TIA1 and G3BP interact with the ternary complex to form SGs.^[^ ^29^ ^]^ In this study, we found that in EAE, neuronal TIA1 expression was significantly upregulated and participated in the formation of G3BP1^+^ SGs (Figure 1). In *Tia1* ^Nestin^‐CKO EAE mice, SGs were reduced (Figure 5A–D,F–I), and p‐eIF2α was also reduced (Figure 5A,E), indicating that TIA1 mediated the formation of SGs in spinal neurons of EAE mice. Evidence indicates that SGs are supposed to be transient structures, and after recovery from stress or stress adaptation, SGs are cleared based on autophagy.^[^ ^30^, ^31^ ^]^ When chronic and severe stress persists, SGs remain in the cell for prolonged periods and facilitate the aggregation of pathological proteins. Within SGs, RBPs demonstrate a marked tendency for abnormal phase separation, resulting in the formation of non‐dynamic hydrogels that are more susceptible to further aggregation into ultra‐stable structures. Furthermore, the high local concentration of proteins within SGs enhances the likelihood of amyloid protein interactions. As a result, this process promotes the continuous formation of pathological oligomers and fibrils, which ultimately induce cytoplasmic neurotoxicity.^[^ ^32^, ^33^ ^]^ Additionally, mutations in proteins such as VCP, optineurin, p62, and ubiquitin‐2 impaired autophagy and hindered abnormal SG clearance, thereby exacerbating disease progression.^[^ ^31^ ^]^ In the present study, we found that SGs persistently existed in the cytoplasm of EAE mice without disassembly, while they were significantly reduced in *Tia1* ^Nestin^‐CKO mice (Figure 5F–I). Similarly, TIA1 knockdown in primary neurons and N2a cells significantly reduced the accumulation of SGs(Figures S4G–I,S5C,K, Supporting Information). LC3, one of the key proteins in the autophagy process, shows an increase, indicating the enhancement of autophagy.^[^ ^34^ ^]^ Interestingly, as expected, we found that in *Tia1* ^Nestin^‐CKO EAE mice, autophagy dysfunction was alleviated and chronic persistent SGs were reduced. The reduction in chronic persistent SGs may result either from a decrease in SG formation or from enhanced autophagic activity leading to increased SG degradation, thereby alleviating neuronal loss and axonal damage. Hence, reversing the irreversible solid‐state of SGs may preserve their reversible liquid properties or facilitate SG disassembly and autophagic clearance, potentially benefiting the treatment of neurodegenerative diseases.^[^ ^35^, ^36^ ^]^

To investigate the underlying mechanisms for enhanced autophagy and attenuated inflammation in *Tia1* ^Nestin^‐CKO EAE mice, we performed RNA sequencing (Figure 6A,B). The sequencing data revealed a notable downregulation in IL‐31RA expression following the knockout of TIA1. T cell reactivation is essential for the development of CNS pathology, and the cytokines derived from this process play a central role in MS pathology.^[^ ^37^ ^]^ IL‐31, a member of the IL‐6 cytokine family, is primarily produced by activated CD4^+^ T cells and is traditionally viewed as an inflammation‐related factor due to its association with chronic inflammation and immune responses. Elevated levels of IL‐31 are indicative of a high degree of inflammation in the disease.^[^ ^38^ ^]^ IL‐31 activates IL‐31RA in neurons, thereby promoting inflammation, allergy, and immune defense.^[^ ^39^, ^40^, ^41^ ^]^ In our study, an upregulation of IL‐31 expression was observed in the spinal cord of EAE mice, along with an increased expression of IL‐31RA in neurons (Figure 6C–L). Simultaneously, we examined the expression of IL‐31 in the cerebrospinal fluid of MS patients. Interestingly, preliminary results indicated that IL‐31 was upregulated in the cerebrospinal fluid of MS patients compared to normal individuals. However, the clinical samples collected in this study are currently limited, and additional samples will be collected for further investigation in the future. Notably, the expressions of both IL‐31 and IL‐31RA were markedly diminished by TIA1 knockout, suggesting that TIA1 may promote EAE in the CNS via upregulating IL‐31/IL‐31RA. IL‐31 influences the surrounding cells by activating multiple signaling pathways. Previous studies have reported that IL‐31, upon activating the IL‐31RA/OSMR complex, triggered p‐ PI3K, which consequently led to a marked increase in p‐AKT.^[^ ^42^, ^43^ ^]^ Our findings indicated a pronounced activation of the PI3K/AKT pathway in the spinal cord tissue of EAE mice, as evidenced by increased phosphorylation levels (Figure 7A–E). Conversely, in *Tia1* ^Nestin^‐CKO EAE mice, this pathway was markedly inhibited, likely a consequence of the reduced expression of IL‐31/IL‐31RA.

The activation of the PI3K/AKT pathway is known to impede autophagy.^[^ ^44^, ^45^ ^]^ Therefore, the suppression of the PI3K/AKT pathway, through the downregulation of IL‐31/IL‐31RA, appeared to restore the disrupted autophagy in *Tia1* ^Nestin^‐CKO EAE mice, leading to the gradual degradation of persistent SGs. Additionally, TIA1 knockdown in N2a cells treated with sodium arsenite inhibited the PI3K/AKT pathway, thereby promoting autophagy and facilitating the clearance of SGs and ROS (Figure S5C–L). The PI3K/AKT signaling pathway is closely associated with NF‐κB activation. A marked reduction in NF‐κB expression was observed in *Tia1* ^Nestin^‐CKO EAE mice (Figure 7A,F,G). NF‐κB is crucial in the progression of diseases within the CNS, particularly in glial cells. Its activation has been detected in various cell types, including astrocytes, oligodendrocytes, microglia and infiltrating macrophages, both in the brains of MS patients and the spinal cords of EAE mice.^[^ ^46^, ^47^ ^]^ The reduced expression of NF‐κB in *Tia1* ^Nestin^‐CKO EAE mice, relative to standard EAE mice, may account for the observed decrease in glial cell proliferation and inflammatory cell infiltration. To further substantiate our hypothesis, we administered 3‐MA, a selective inhibitor of PI3K, to EAE mice.^[^ ^48^ ^]^ Following administration, there was a marked improvement in neuroinflammation and demyelination symptoms in these mice (Figure 8H–K,P–V), thereby suppressing the pivotal role of the PI3K/AKT signaling pathway in the advancement of EAE.

We noticed that *Nestin‐cre* mice could not accurately delineate the function of neuronal TIA1 in EAE due to its expression of cre recombinase in neural stem cells regulated by the Nestin promoter. Therefore, we cannot overlook the role of TIA1 in neural stem cells and their derivatives, including oligodendrocytes and astrocytes.^[^ ^49^ ^]^ Thus, employing *Synapsin I‐Cre* transgenic mice, which exhibit greater specificity, would be preferable for studying neuronal function.^[^ ^50^ ^]^ Several other studies highlight the importance of TIA1 and SGs in the survival and function of oligodendrocytes and astrocytes, which is particularly relevant to EAE and MS.^[^ ^51^, ^52^, ^53^ ^]^ Future studies focusing on the role of TIA1 and SGs in specific cells may aid in understanding the pathogenic mechanisms caused by different cells in MS.

In summary, we present evidence for a novel signaling mechanism of TIA1‐mediated alternation of SGs dynamics and autoimmune response in the MS. We demonstrated that TIA1 knockout in the CNS inhibited the PI3K/AKT signaling pathway in neurons via reduction of IL‐31RA, which further enhanced autophagy and decrease chronic persistent SGs that caused alleviation of neurodegenerative lesions such as demyelination in neurons. Moreover, NF‐κB protein and various immune cytokines were significantly decreased, indicating the immune microenvironment improvement in EAE. Our findings uncover an unacknowledged role of neuronal TIA1 in promoting axonal injury, demyelination, and neuroinflammation, elucidating potential regulatory pathways that could aid in the development of novel therapies for MS.

## 4 Experimental Section

### Animals

*Tia1* ^Nestin^‐CKO mice were generated by crossing the floxed *Tia1* allele (*Tia1* ^f^^/^^f^) mice (Shanghai Biomodel Organism Science & Technology Development Co., Ltd) with Nestin‐Cre transgenic mice (from The Jackson Laboratory). The Nestin‐Cre mice express Cre recombinase specifically in neural stem cells under the control of the Nestin promoter, allowing for conditional knockout of genes in neural stem cells and their derivatives, including neurons, astrocytes, and oligodendrocytes. These mice were based on the C57BL/6 background and genotyped by PCR. The primer sequences used in this study are listed in Table S1 (Supporting Information). All animal experiments strictly followed the guidelines of the Laboratory Animals Ethics Committee of Hangzhou Normal University (HSD20220106).

### EAE Model and Treatment

The EAE model utilized in this study was previously described.^[^ ^54^ ^]^ In brief, female mice with body weights ranging from 15 to 20 g were anesthetized and subsequently immunized subcutaneously with 200 ng of emulsified MOG35‐55 peptide (dissolved in double‐distilled water, HY‐P1240A, MCE) or PBS in a 1:1 ratio with Complete Freund's Adjuvant (CFA) containing 8 mg mL^−1^ mycobacterium tuberculosis (H37RA strain, Difco, USA). Additionally, 300 ng of pertussis toxin (PTX, Sigma, dissolved in PBS) was administered intraperitoneally concurrent with the initial immunization and then again two days later. The body weight of each mouse was meticulously recorded, and EAE scores were evaluated based on a previously published five‐point scoring criterion (1, tail weakness or paralysis; 2, faltering gait with hind limb weakness; 3, severe leg weakness; 4, complete limb paralysis; 5, death) from day 0 to day 21 post‐immunization.^[^ ^55^ ^]^

For the inhibition of PI3K in this model, mice were treated daily with 3‐methyladenine (3‐MA, 10 mg k^−1 ^g, Sigma, M9281) following EAE induction. All mice were randomly allocated to either control or experimental groups. A double‐blind evaluation was employed to assess experimental conditions and genotypes, ensuring the objectivity and reliability of the study outcomes.

### Primary Neuron Culture

For primary neuron cultures, pregnant *Tia1* ^f^^/^^f^ and *Tia1* ^N^ ^e^ ^s^ ^t^ ^i^ ^n^‐CKO mice were euthanized.

The fetal mice brains were carefully collected and dissociated with Hank's Balanced Salt Solution (HBSS) medium. After removing the cortex, the hippocampus was dissected from the surface of the brain, chopped, and then incubated with 0.125% trypsin (C0208, Beyotime) at 37 °C for 12 min. After washing with HBSS, cells were resuspended and cultured at 37 °C with 5% CO_2_ in Neurobasal medium (Gibco, 21103‐049) supplemented with 1% penicillin/streptomycin (Gibco, 15140‐122), 2% B27 (Gibco, 17 504 044), 2 mM Glutamine (Invitrogen, 25030–081) and 50 ng mL^−1^ BDNF (PeproTech, 450‐02) as described previously.^[^ ^56^ ^]^ The culture media were changed every 2–3 days. To stress the neurons, primary neurons were treated with 0.5 mm sodium arsenite (Sigma, S7400) for 1 h.

### Neuro‐2a (N2a) Cell Culture

The Neuro‐2a (N2a) cell line (to mimic neuronal characteristics in vitro, Cat No. FH0424, provided by Shanghai Fuheng Biotechnology Co., Ltd.) was first cultured in Dulbecco's Modified Eagle's Medium (DMEM; Gibco, Carlsbad, CA, USA) supplemented with 10% fetal bovine serum (FBS; Gibco) at 37 °C in a humidified incubator (5% CO₂, 95% air). Cells were treated with 0.5 mm sodium arsenite (Sigma, S7400) for 1 h to induce stress. To inhibit lysosomal autophagy, cells were treated with 100 µm chloroquine (MCE, HY‐17589A) during the stress treatment. ROS was measured using CellROX Deep Red (Thermo Fisher Scientific, C10422).

### RNA Interference Experiments

The siRNAs targeting TIA1, along with negative controls, were designed and synthesized by Sigma‐Aldrich. Cell transfection was performed according to the manufacturer's instructions using Lipofectamine 3000 (Thermo Fisher, USA). Following transfection, cells were harvested for RNA and protein validation, as well as for functional assays. The sequence of the siRNA oligonucleotide was: 5′‐GCTCTAATTCTGCAACTCTTT‐3′.

### Hematoxylin‐Eosin (HE) Staining

After anesthetization, Mice underwent perfusion with 4% paraformaldehyde (PFA) and 0.1 m PBS. Subsequently, their spinal cords were immersed in 4% PFA for 24 h and then transferred to a 30% sucrose solution until full submersion. Thereafter, the spinal cords were embedded in an optimal cutting temperature (OCT) compound and frozen at −20 °C. These embedded tissues were sectioned into 20 µm slices using a Thermo freezing microtome (CryoStar NX50, Thermo), and the sections were carefully mounted onto adhesive glass slides. The sections underwent hematoxylin staining for 5 min for histological analysis, followed by a thorough rinse in double‐distilled water. They were then stained with eosin for 10 s and sequentially dehydrated using 75%, 95%, and 100% ethanol, each for 1 min. Finally, the sections were cleared in xylene for 10 min and permanently mounted using a neutral resin. Microscopic images of the stained sections were captured using a SLIDEVIEW VS200 microscope (Olympus), and quantitative analysis of these images was conducted using Image J software (Media Cybernetics, Bethesda, MD, USA).

### Nissl's Staining

Spinal cord sections, prepared as per previously established protocols, were processed for histological analysis.^[^ ^57^ ^]^ The 20 µm‐thick sections were incubated in 0.1% cresyl violet solution at room temperature for 6 min, followed by a 5‐min rinse in double‐distilled water. The sections were sequentially dehydrated in 95% and 100% ethanol before being cleared in xylene for 10 min and set in neutral resin. Imaging was performed using a SLIDEVIEW VS200 microscope (Olympus), and the acquired images were quantitatively analyzed with Image J software.

### Luxol Fast Blue (LFB) Staining

The 20‐µm‐thick spinal cord sections underwent overnight staining in LFB staining solution (Solarbio, G3242) at room temperature. Following the staining, the sections were rinsed in 95% ethanol and subsequently washed with distilled water. For enhanced contrast, the sections were counterstained in Luxol differentiation solution for 15 s, followed by a 30‐second differentiation in 70% ethanol, ensuring a clear distinction between gray and white matter.

Subsequently, the sections were dehydrated through sequential immersion in 95% and 100% ethanol, cleared in xylene, and sealed with resinene. Demyelination in the LFB‐stained sections was quantitatively assessed on a scale: 0 indicating no demyelination, 1 for moderate, and 2 for severe demyelination.^[^ ^58^ ^]^ Images were captured using a SLIDEVIEW VS200 microscope (Olympus) and were analyzed using Image J software.

### Immunostaining

After three washes with PBS, the spinal cord slices were fixed in 4% PFA for 30 min, followed by incubation in 5% BSA containing 0.3% Triton X‐100 for 1 h. This was followed by an overnight incubation at 4 °C with various primary antibodies. The subsequent day, after three PBS washes, the sections were incubated with corresponding secondary antibodies and DAPI in 5% BSA for 1 h at room temperature. The primary antibodies included mouse anti‐NeuN (1:500, ab104224, abcam), mouse anti‐GFAP (1:500, MAB360, Millipore), rabbit anti‐CD45 (1:500, ab10558, abcam), rabbit anti‐NeuN (1:500, 94 403, CST), rabbit anti‐NF (1:500, ab8135, abcam), mouse anti‐MBP (1:500, ab62631, abcam), rabbit anti‐TIA1 (1:500, AB140595, abcam), rabbit anti‐PH3 (1:500, ab14955, abcam), rabbit anti‐Aldh1L1 (1:200, ab177483, abcam), rabbit anti‐GAP43 (1:500, ab16053, abcam), rabbit anti‐Iba1 (1:200, ab153696, abcam), rabbit anti‐Ki67 (1:200, #9129, CST), rabbit anti‐G3BP1 (1:500, 13 057, Proteintech), rabbit anti‐G3BP2 (1:500, 16 276, Proteintech), rabbit anti‐IL‐31 (1:200, DF8986, Affinity), mouse anti‐IL‐31RA (1:100, sc‐515465, Santa), rabbit anti‐LAMP1 (1:100, AB208943, abcam), anti‐SQSTM1/p62 (1:500, #23 214, CST) and rabbit anti‐F4/80 (1:100, AB90247, abcam). Secondary antibodies included donkey anti‐mouse Alexa Fluor488, donkey anti‐rabbit Alexa Fluor546, donkey anti‐rabbit Alexa Fluor488 (1:1, 000, A21206, Invitrogen), donkey anti‐goat Alexa Fluor488 (1:1, 000, A11055, Invitrogen) and donkey anti‐mouse Alexa Fluor546 (1:1, 000, A10036, Invitrogen). Microscopic imaging was performed at room temperature using a SLIDEVIEW VS200 microscope (Olympus) and analyzed using Adobe Photoshop and ImageJ software.

### Western Blotting

Mice were anesthetized with intraperitoneal injection of tribromoethanol. The lumbar spinal cords were collected following cardiac perfusion with PBS, then homogenized three times in a lysis solution consisting of RIPA buffer (P0013b, Beyotime), 100 mm phenylmethylsulfonyl fluoride (PIC) for 45 s, and incubated at 4 °C for 30 min. After centrifugation for 20 min, the supernatant was combined with 5× loading buffer and then heated at 100 °C for 10 min to prepare protein samples. These samples were then subjected to separation using 8%, 10%, and 12% sodium dodecyl sulfate‐polyacrylamide gel electrophoresis, followed by transfer to PVDF membranes (Pierce Chemical Company, Illinois, USA). The membranes were blocked with 1× protein‐free rapid blocking buffer for 20 min at room temperature, then incubated overnight at 4 °C with various primary antibodies. The primary antibodies included mouse anti‐β‐actin (1:10, 000, A5316, Sigma‐Aldrich), mouse anti‐MBP (1:1, 000, ab62631, abcam), mouse anti‐IL‐31RA (1:1, 000, sc‐515465, Santa Cruz), mouse anti‐GAPDH (1:5, 000, #T0004, Affinity), rabbit anti‐p‐eIF2α (1:1, 000, 28 740, Proteintech), rabbit anti‐G3BP1 (1:1, 000, 13 057, Proteintech), rabbit anti‐G3BP2 (1:1, 000, 16 276, Proteintech), rabbit anti‐CC3 (1:1, 000, #9661, CST), rabbit anti‐TIA1 (1:1, 000, 12 133, Proteintech), rabbit anti‐p‐PI3K (1:1, 000, 17366S, CST), rabbit anti‐PI3K (1:1, 000, 4257, CST), rabbit anti‐p‐AKT (1:1, 000, 13 038, CST), mouse anti‐AKT (1:1, 000, 2920, CST), rabbit anti‐LC3 (1:1, 000, 4108S, CST), rabbit anti‐IL‐31 (1:1, 000, DF8986, Affinity), rabbit anti‐SQSTM1 (1:1, 000, #23 214, CST).

The following day, after washing with TBST for 30 min, the membranes were incubated with horseradish peroxidase‐conjugated secondary antibodies (goat anti‐mouse IgG‐HRP, 1:5 000, #31 460, Pierce; goat anti‐rabbit IgG‐HRP 1:5  000, #31 420, Pierce) for 1 h at room temperature. Detection (GelViev 6000Plus, BLT) of the protein bands was achieved using an ECL detection kit (1 705 061, Bio‐Rad, USA), and the resulting images were analyzed using Image J software.

### Electron Microscopy

Electron microscopy was performed following a previously established protocol.^59^ ^]^ The extracted tissues were initially fixed overnight in 2.5% glutaraldehyde (sourced from Guoyao Chemical Reagent Co., Ltd.). Post‐fixation, the samples underwent a series of washes with PBS, followed by a secondary fixation in 1% osmium tetroxide (SPI‐CHEM). After another set of PBS washes, the samples were dehydrated through a gradient of alcohol concentrations. Subsequently, they were transitioned to absolute acetone (Sinopharm Chemical Reagent Co., Ltd) and embedded in a mixture of absolute acetone and Spurr resin (SPI‐CHEM), followed by a curing process at 70 °C for over 9 h. Sectioning of the samples was done using a LEICA EM UC7 ultratome. The sections were then stained with uranyl acetate and alkaline lead citrate (Sinopharm Chemical Reagent Co., Ltd) and examined using a Hitachi Model H‐7650 transmission electron microscope.

### Nuclear‐Cytoplasmic Fractionation

The nuclear‐cytosol extraction kit (EX2660, Solarbio) was used to separate the nuclear and cytoplasmic proteins of spinal cord tissues following the manufacturer's instructions. The fractions were analyzed by western blot with specific antibodies.

### Quantitative Reverse Transcription Polymerase Chain Reaction (qRT‐PCR)

Total RNA extraction was carried out using the RNA‐Quick Purification Kit (YISHAN Biotechnology, ES‐RN001) according to the manufacturer's protocol. The quantification of RNA was performed using the Thermo Scientific NanoDrop One. The cDNA DyNAmo Kit (Vazyme, R211‐01/02) was employed for cDNA synthesis. qPCR was conducted using SYBR Green PCR Master Mix (Vazyme, Q511‐02/03). The real‐time PCR cycling conditions were programmed as follows: an initial step at 95 °C for 15 min, followed by 40 cycles of denaturation at 94 °C for 15 s, annealing at 56 or 60 °C for 30 s, and a final extension at 72 °C for 30 s. Post‐amplification, a melting curve analysis was performed to verify the primer specificity. The Ct values obtained were normalized against the corresponding β‐actin control Ct values. The primer sequences used in this study are listed in Data 1 (Supporting Information).

### RNA Sequencing and Functional Enrichment Analysis

The RNA sequencing was performed as the previous report.^[^ ^49^ ^]^ RNA sequencing was performed by the Novogene Bioinformatics Institute (Beijing, China). Total RNA was extracted from the spinal cords of *Tia1* ^Nestin^‐CKO and *Tia1* ^f/f^ EAE mice using Trizol (Invitrogen) according to the manufacturer's instructions. RNA purity was assessed using the Nanodrop, and each sample had an A260:A280 ratio > 1.8 and an A260:A230 ratio > 2.0. Total amounts and integrity of RNA were assessed using the RNA Nano 6000 Assay Kit of the Bioanalyzer 2100 system (Agilent Technologies, CA, USA). AMPure XP system (Beckman Coulter, Beverly, USA) was used to purify the library fragments and 370–420 bp cDNA was selected. Following PCR amplification, the PCR product was purified using AMPure XP beads, resulting in the final library. After the library was constructed, it was initially quantified by Qubit2.0 Fluorometer and diluted to 1.5 ng µl^−1^, then the insert size of the library was detected by Agilent 2100 bioanalyzer. After that, qRT‐PCR was used to accurately quantify the effective concentration of the library to ensure the quality. Sequencing was performed using the Illumina NovaSeq 6000, and the end reading of the 150 bp pairing was generated.

Clean data (clean reads) were obtained by removing reads containing adapter, reads containing N base and low‐quality reads from raw data. The reference genome index was constructed with Hisat2 (v2.0.5), and paired‐end clean reads were aligned to this reference genome using Hisat2. Subsequently, FeatureCounts (v1.5.0‐p3) was employed to quantify the number of reads mapped to each gene, followed by the calculation of the expected number of Fragments Per Kilobase of transcript sequence per Million base pairs sequenced (FPKM) for each gene. Differential expression analysis of two groups was performed by using the DESeq2 R package (1.20.0). The threshold for statistical significance in these analyses was set at a *p*‐value less than 0.05. GO enrichment analysis relied on all differentially expressed genes with a *p*‐value < 0.05, which were considered significantly enriched.

### Flow Cytometry Analysis

Spleens were harvested from mice, submerged in staining buffer (serum, EDTA, PBS), crushed using the back of needles, and filtered into tubes using 200‐mesh filter paper. The resulting mixture was centrifuged to collect the cells. Subsequently, red blood cells were lysed using ACK lysis buffer for 5 min at 37 °C, followed by another round of centrifugation to collect the cells. Collected cells were re‐suspended with the staining buffer, and the appropriate amount of cell suspension was centrifuged and collected, then were added with stimulation factors (PMA, ionomycin, BFA, 1640 cell culture medium) and placed in cell incubators for 4 h. The collected cells after centrifugation were stained with surface markers at room temperature for 15 min, and viability was assessed with zombie aqua. These cells were then washed with staining buffer once, obtained by centrifugation, and fixed by the Ic fixation buffer at room temperature for 20 min. After termination with permeabilization buffer, the collected cells were stained for intracellular markers at room temperature for 1 h. Afterward, staining was halted using the permeabilization buffer, and the staining buffer was added once the supernatant was discarded. Stained cells were analyzed on a BD FACSDiva Fortessa (Zhejiang University Flow Cytometry Shared Facility), and data were analyzed using FlowJo (v10.8.1).

The flow cytometry antibodies, their source, and dilution information were as followed: Zombie Aqua Fixable Viability Kit (1:1, 000, 423 101, BioLegend); surface markers: Brilliant Violet605 anti‐mouse CD45 (30‐F11) (1:500, 103 140, BioLegend), PerCP/Cyanine5.5 anti‐mouse CD4 (GK1.5) (1:300, 100 434, BioLegend), FITC anti‐mouse/human CD11b (M1/70) (1:300, 101 206, BioLegend), PE anti‐mouse F4/80 (BM8) (1:300, 123 110, BioLegend); intracellular markers: PE/Cyanine7 anti‐mouse IFNγ (XMG1.2) (1:200, 505 826, BioLegend), APC anti‐mouse IL17 (TC11‐18H10.1) (1:200, 506 916, BioLegend).

### Cerebrospinal Fluid (CSF) Sample

The CSF of the MS patient was obtained from the Department of Neurology at the First Affiliated Hospital of Wenzhou Medical University. The patients experienced recurrent limb stiffness and weakness for over 10 years, with a recurrence occurring more than 2 months ago. Considering a diagnosis of “MS”, a lumbar puncture was performed to confirm the diagnosis, which indeed revealed multiple sclerosis. CSF was obtained during the clinical diagnosis and treatment process via lumbar puncture, and the remaining portion was stored frozen at −80 °C after clinical testing. Regarding the control group, CSF samples were acquired from patients admitted to the Department of Neurology at the First Affiliated Hospital of Wenzhou Medical University. These patients presented with dizziness as their primary complaint, without exhibiting additional clinical signs or symptoms. Routine blood tests and CSF examinations revealed no abnormalities, thereby ruling out severe neuroinflammatory conditions. Following imaging assessments, these patients were diagnosed with radiologically isolated syndrome. CSF samples were collected via lumbar puncture as part of their clinical diagnosis and treatment regimen, and subsequently stored at −80 °C. Approval for this study was obtained from the Ethics Committee in Clinical Research at the First Affiliated Hospital of Wenzhou Medical University (KY2024‐R106).

### Data Analysis and Statistics

The densities of astrocytes (GFAP^+^) and microglia (Iba1^+^) were calculated by counting them across whole spinal cord cross‐sections, then dividing by the areas of the spinal cord slices analyzed using Image J All data represent the mean ± SEM from at least three independent experiments. Statistical analyses were conducted using GraphPad Prism 5 and Image J software. Both Student's *t*‐test and ANOVA were used for statistical evaluations, supplemented with Bonferroni's post‐hoc tests. A *p*‐value of less than 0.05 was considered statistically significant. Additional details were found in the Figure legends accompanying this study.

## Conflict of Interest

The authors declare no conflict of interest.

## Author Contributions

X.H., L.J., and Z.F. contributed equally to this work. X.Z., Z.H., and J.L. managed and designed the overall study. X.H., L.J., and Z.F. performed experiments. X.H. wrote the manuscript. Y.T. and S.G. performed Flow cytometry analysis. J.L., Y.H., and Y.D. collected cerebrospinal fluid samples. X.H., L.J., Z.F., Y.W., Y.Z., J.Z., and D.X. performed the statistical analysis and checked the manuscript.

## Supporting information



Supporting Information

## Data Availability

Research data are not shared.
