# Supplementary material for: TIA1‐Mediated Stress Granules Promote the Neuroinflammation and Demyelination in Experimental Autoimmune Encephalomyelitis through Upregulating IL‐31RA Signaling
Source: Adv Sci (Weinh). 2025 Jan 13;12(14):2409086. doi: 10.1002/advs.202409086 (PMC11984900; doi:10.1002/advs.202409086)
Supplement: Supplementary file 1 — Supporting Information [file ADVS-12-2409086-s001.docx]

**Supplementary massterials**

**
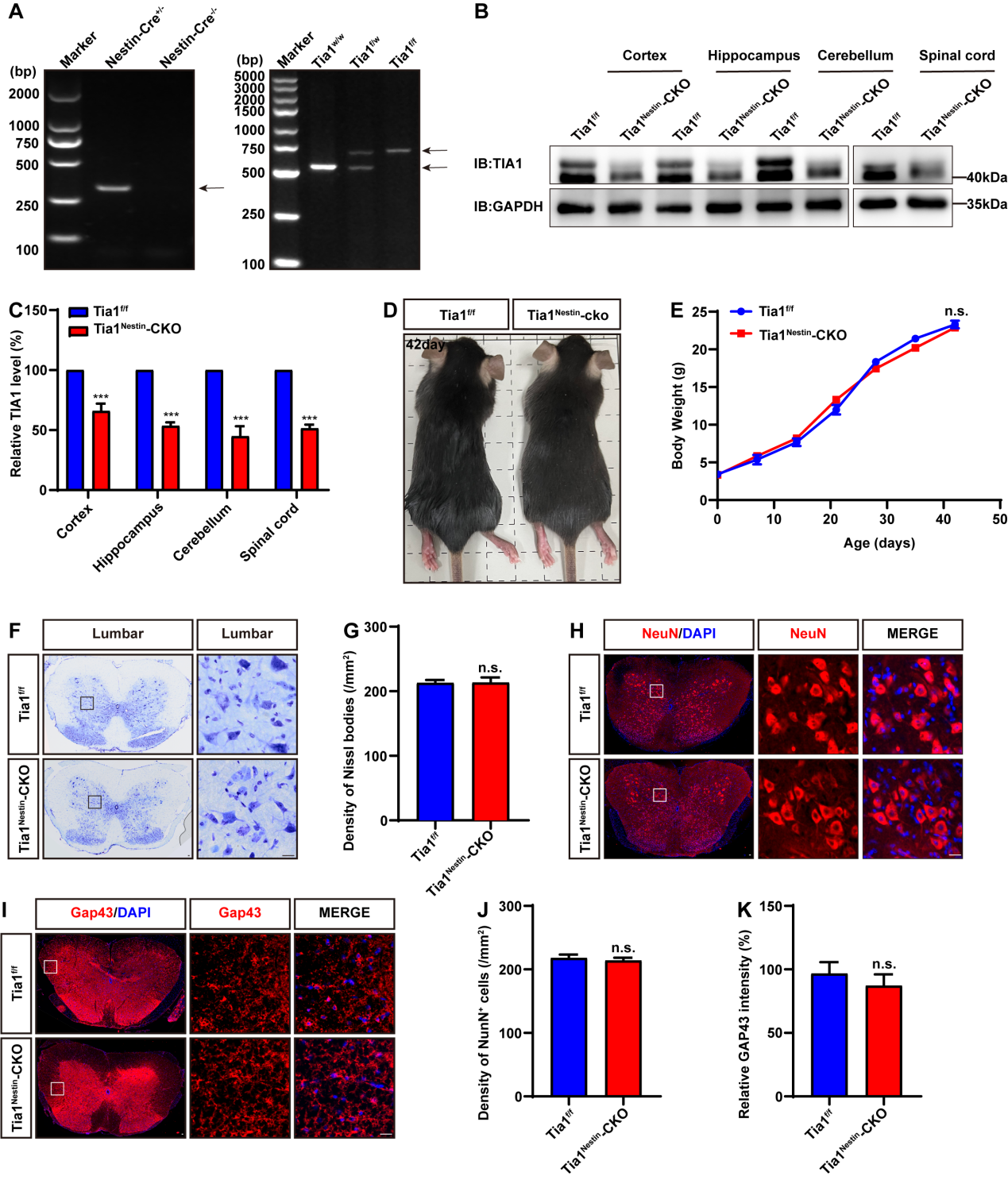
**

**Figure S1. TIA1 knockout in the CNS didn’t affect spinal cord development in mice.** (**A**) Genotyping identification of *Tia1*^Nestin^-CKO mice. (**B**) Western blot analysis of TIA1 expression in the cortex, hippocampus, cerebellum and spinal cord of 8-week-old *Tia1*^f/f^ mice and *Tia1*^Nestin^-CKO mice. (**C**) Quantitative analysis of the relative TIA1 levels as shown in (**B**) (normalized to control mice, n = 4 mice per group, paired t-test). (**D**) Representative images of the growth morphology of 6-week-old *Tia1*^f/f^ and *Tia1*^Nestin^-CKO mice. (**E**) The weight of *Tia1*^f/f^ mice and *Tia1*^Nestin^-CKO mice ranged from 0 to 42 days (n = 5 mice per group, two-way ANOVA with Bonferroni’s post-tests). (**F, H**) Typical images of Nissl staining (**F**) and NeuN^+^ immunostaining (**H**) in the lumbar spinal cords of 8-week-old *Tia1*^f/f^ mice and *Tia1*^Nestin^*-*CKO mice. (**G, I**) Quantitative analysis of the density of Nissl bodies as shown in (**F**) (**G**, n = 4 sections from 4 mice per group), and the density of NeuN^+^ cells as shown in (**H**) (**I**, n = 4 sections from 4 mice per group). (**J**) Immunostaining of GAP43 (red) in spinal cords of *Tia1*^f/f^ mice and *Tia1*^Nestin^-CKO mice. (**K**) Quantitative analysis of the relative GAP43 expression as shown in (**J**) (n = 4 sections from 4 mice per group). Scale bars, 20 μm. Data were mean ± SEM. Student’s t-test unless otherwise indicated, n.s., not significant.


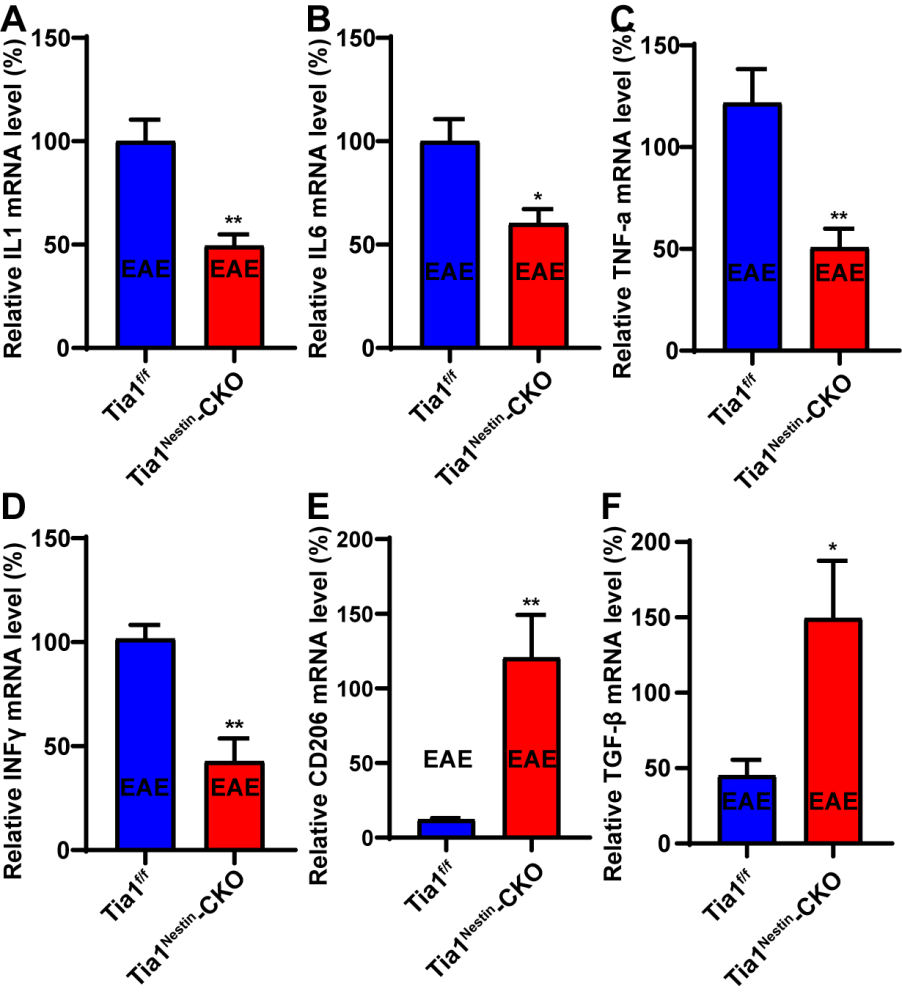


**Figure S2. The pro-inflammatory factors were decreased and the immune-regulatory factors were enhanced in *Tia1*^Nestin^-CKO EAE mice.** (**A**-**F**) qPCR analysis of the relative mRNA level of IL-1 (**A**, n = 6 mice per group), IL-6 (**B**, n = 6 mice per group), TNF-α (**C**, n = 4 mice per group), INFγ (**D**, n = 5 mice per group), CD206 (**E**, n = 4 mice per group) and TGF-β (**F**, n = 5 mice per group) in spinal cords of *Tia1*^f/f^ EAE mice and *Tia1*^Nestin^-CKO EAE mice. Data were mean ± SEM. Student’s t-test, *^*^p < 0.05, ^**^p < 0.01.*


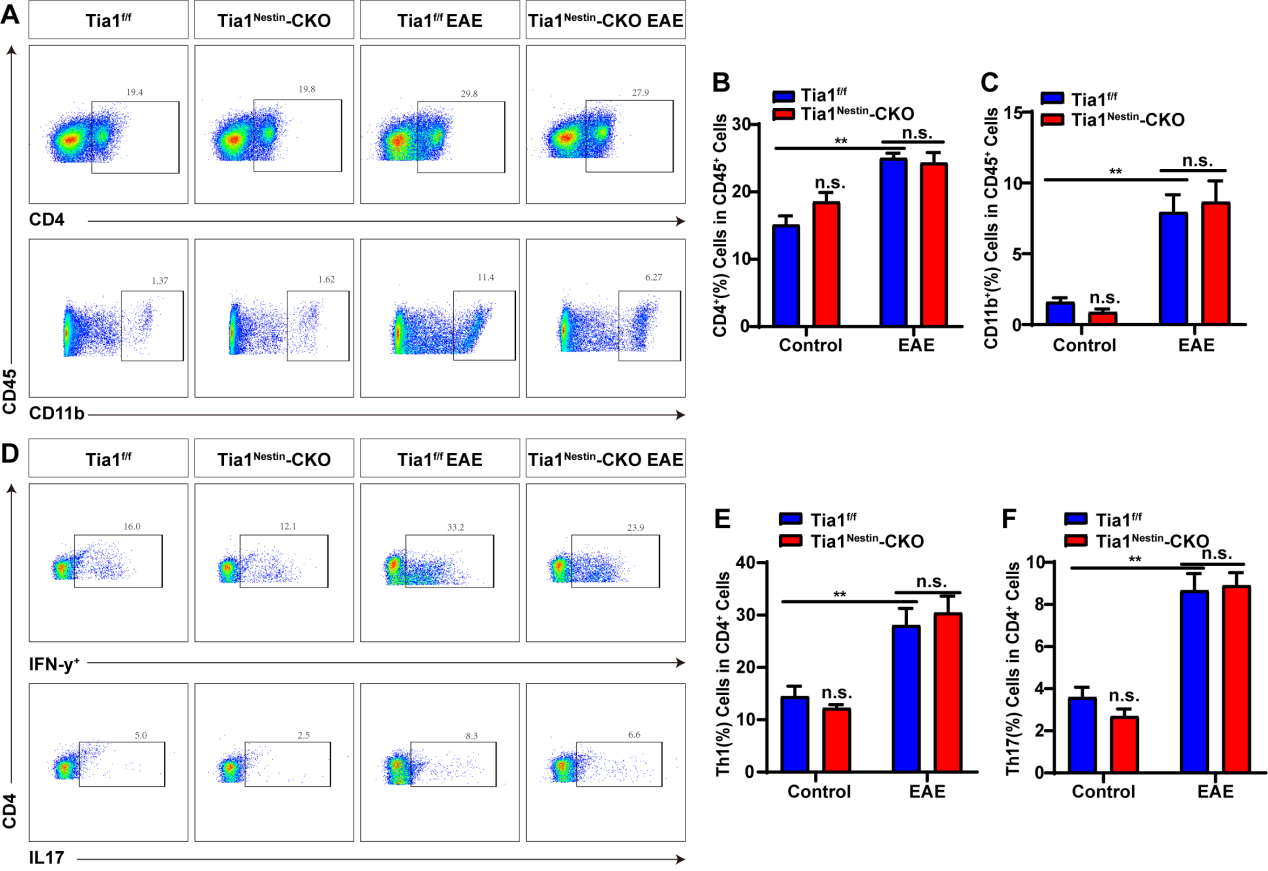


**Figure S3. TIA1 knockout in the CNS didn’t affect the peripheral spleen T cell populations in EAE mice.** (**A**) Flow cytometry analysis of splenic immune cells (including CD45^+^/CD4^+^ T cells and CD45^+^/CD11b^+^ monocytes) in *Tia1*^f/f^ and *Tia1*^Nestin^-CKO mice, as well as in *Tia1*^f/f^ EAE and *Tia1*^Nestin^-CKO EAE mice. (**B-C**) Statistical analysis of the relative cell numbers of CD45^+^ CD4^+^ T cells (**B**) and CD11b^+^ T cells (**C**) from (**A**) (n = 5 mice per group). (**D**) Flow cytometry analysis of Th1 (IFN-γ^+^) and Th17 (IL-17^+^) cells in the spleen of *Tia1*^f/f^ and *Tia1*^Nestin^-CKO mice, and *Tia1*^f/f^ EAE and *Tia1*^Nestin^-CKO EAE mice. (**E-F**) Statistical analysis of the relative cell numbers of Th1 (IFN-γ^+^) cells (**E**, n = 5 mice per group) and Th17 (IL-17^+^) cells (**F**, n = 4 mice per group) from (**D**). Data were mean ± SEM. Two-way ANOVA, *^*^p < 0.05, ^**^p < 0.01.*

*
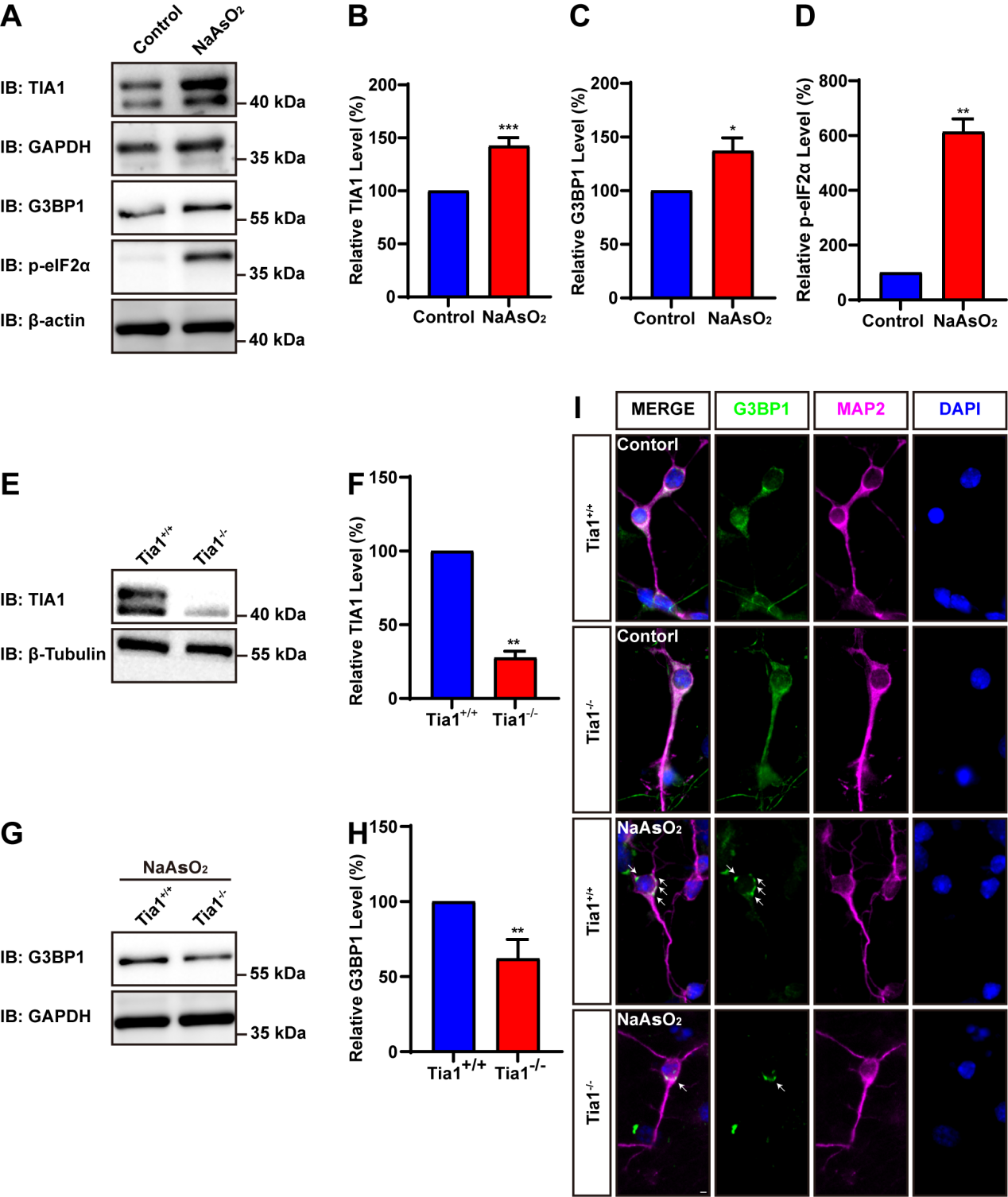
*

**Figure S4. TIA1-mediated stress granule formation in neurons induced by sodium arsenite.** (**A**) Western blot analysis of TIA1, G3BP1 and p-eIF2α expression in primary cultured neurons treated with control and sodium arsenite (NaAsO2). (**B-D**) Quantitative analysis of the relative TIA1 (**B**), G3BP1(**C**) and p-eIF2α levels (**D**) as shown in (**A**) (normalized to control neurons, n =3 per group, paired t-test). (**E**) Western blot analysis of expression of TIA1 in primary cultured Tia1^+/+^ and Tia1^-/-^ neurons. (**F**) Quantitative analysis of relative expression of TIA1 was shown in (**E**) (normalized to Tia1^+/+^, n = 4 per group, paired t-test). (**G**) Western blot analysis of G3BP1 expression in primary cultured Tia1^+/+^ and Tia1^-/-^ neurons treated with sodium arsenite. (**H**) Quantitative analysis of relative expression of G3BP1 was shown in (**G**) (normalized to Tia1^+/+^, n = 4 per group, paired t-test). (**I**) Double immunostaining of G3BP1 (green) and MAP2 (purple) in primary cultured Tia1^+/+^ and Tia1^-/-^ neurons treated with sodium arsenite. Scale bars, 20 μm. Data were mean ± SEM. ^*^*p* < 0.05, ^**^*p* < 0.01.


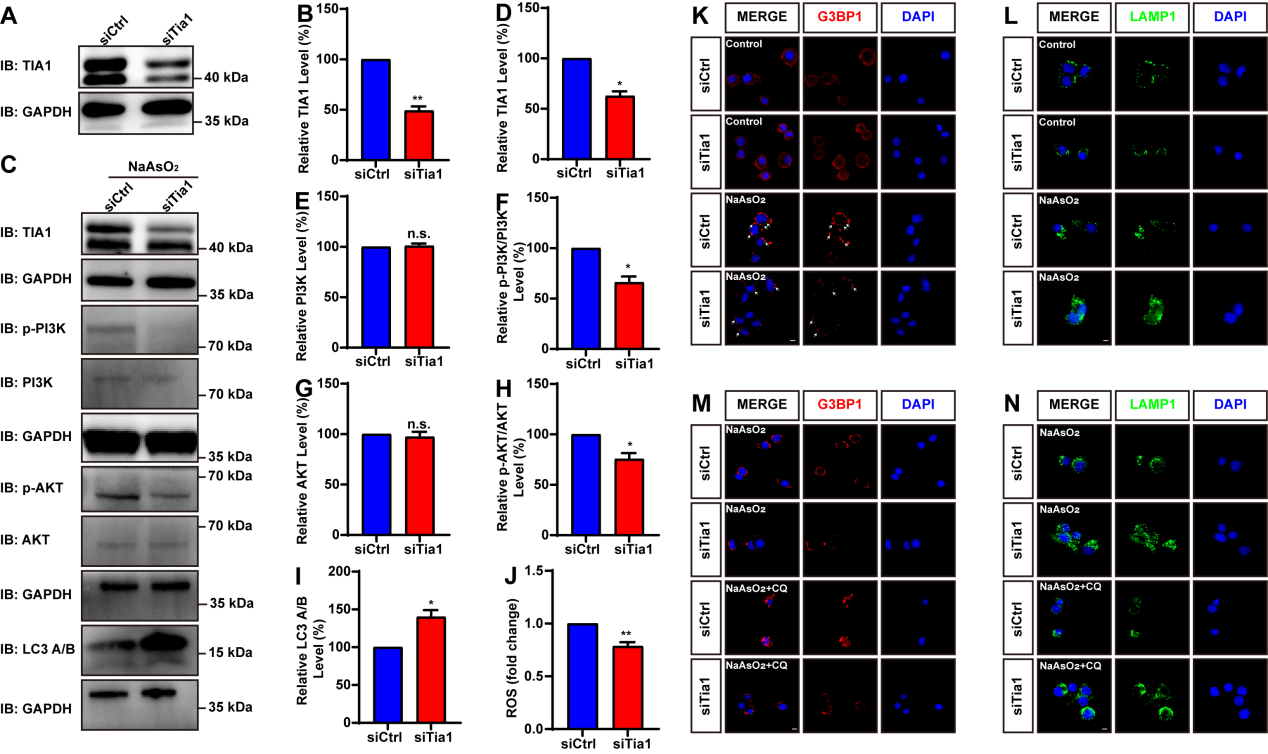


**Figure S5. TIA1 knockdown enhances autophagy and reduces stress granules in N2a cells.** (**A**) Western blot analysis of TIA1 expression in control and siTia1 N2a cell. (**B**) Quantitative analysis of the relative TIA1 as shown in (**A**) (normalized to control neurons, n = 4 per group, paired t-test). (**C**) Western blot analysis of TIA1, p-PI3K, PI3K, p-AKT, AKT and LC3 A/B expression in control and siTia1 N2a cell treated with sodium arsenite. (**D-I**) Quantitative analysis of the relative TIA1 (**D**), PI3K (**E**), p-PI3K/PI3K (**F**), AKT (**G**), p-AKT/AKT (**H**) and LC3 A/B (**I**) as shown in (**C**) (normalized to control neurons, n =4 per group, paired t-test). (**J**) Quantification of ROS accumulation in control and siTia1-treated N2a cells after sodium arsenite treatment. (**K, L**) Immunostaining of G3BP1 (**K**) and LAMP1 (**L**) in control and siTia1 N2a cell treated with sodium arsenite. (**M, N**) Immunostaining of G3BP1 (**K**) and LAMP1 (**L**) in control and siTia1 N2a cell treated with sodium arsenite and CQ. Scale bars, 20 μm. Data were mean ± SEM. ^*^*p* < 0.05, ^**^*p* < 0.01.

**Table S1: Primer sequences**

| **Primer** | **Sequence** |
| --- | --- |
| TIA1-F | 5’-GAGGCATCAGAATTGTTTTAGTG-3’ |
| TIA1-R | 5’-GAGATTCTGCGGGGCGATAG-3’ |
| Nestin-Cre-F | 5’-CAGCATTGCTGTCACTTGGTC-3’ |
| Nestin-Cre-R | 5’-ATTTGCCTGCATTACCGGTCG-3’ |
| IL31RA-F | 5’-TCAGCAGACGAATCAATACAGC-3’ |
| IL31RA-R | 5’-TCGCTCAACACTTTGACTTTCT-3’ |
| IL31-F | 5’-TCCTGATGTTCCCAACCCTG-3’ |
| IL31-R | 5’-TTAGGACCACGTCTTCTGTGT-3’ |
| IL1β-F | 5’-AAGGGCTGCTTCCAAACCTTTGAC-3’ |
| IL1β-R | 5’-ATACTGCCTGCCTGAAGCTCTTGT-3’ |
| IL6-F | 5’-ATCCAGTTGCCTTCTTGGGACTGA-3’ |
| IL6-R | 5’-TAAGCCTCCGACTTGTGAAGTGGT-3’ |
| TNFα-F | 5’-TCTCATGCACCACCATCAAGGACT-3’ |
| TNFα-R | 5’-ACCACTCTCCCTTTGCAGAACTCA-3’ |
| CD206-F | 5’-TCAGCTATTGGACGCGAGGCA-3’ |
| CD206-R | 5’-TCCGGGTTGCAAGTTGCCGT-3’ |
| TGFβ-F | 5’-GACATCAAAAGATAACCACTC-3’ |
| TGFβ-R | 5’-GACATCAAAAGATAACCACTC-3’ |
| IFNγ-F | 5’-ATGAACGCTACACACTGCATC-3’ |
| IFNγ-R | 5’-CCATCCTTTTGCCAGTTCCTC-3’ |
| β-actin-F | 5’-GGCACCACACCTTCTACAATG-3’ |
| β-actin-R | 5’-GGGGTGTTGAAGGTCTCAAAC-3’ |
